# GmNFYA14 negatively regulates soybean seed oil content by interacting with GmWRI1a

**DOI:** 10.3389/fpls.2026.1916663

**Published:** 2026-10-28

**Authors:** Han Gou, Xi Li, Shuxin Chai, Meiling Chen, Xiaochao Chen, Yunhua Yang, Huan Du, Bao-Jian Ding, Han Xing, Ping Deng, Fengjie Yuan, Bei Liu

**Affiliations:** 1 Xianghu Laboratory, Hangzhou, China; 2 College of Agriculture, Nanjing Agricultural University, Nanjing, China; 3 Zhejiang Academy of Agricultural Sciences, Hangzhou, China

**Keywords:** GmNFYA14, GmWRI1, molecular mechanism, oil content, soybean

## Abstract

**Introduction:**

High oil content determines the commercial value of soybean, so the mechanisms that regulate oil accumulation are important targets for quality improvement. *GmWRI1a* is a core regulator of lipid biosynthesis in soybean, but the proteins that cooperate with it are still poorly defined.

**Methods:**

Yeast two‑hybrid screening using truncated GmWRI1a as bait identified GmNFYA14. Multiple *in-vitro* and *in-vivo* protein interaction assays verified the physical interaction between GmNFYA14 and GmWRI1a. We generated *GmNFYA14* overexpression and CRISPR/Cas9 knockout soybean lines to analyze seed storage components, and combined dual‑luciferase, EMSA and RNA‑seq assays to dissect the regulatory mechanism.

**Results:**

Overexpression of *GmNFYA14* reduced seed oil content by 2.96–4.13%, total fatty acid content by 9.90–10.78% and triacylglycerol content by 6.32–8.88%, while increasing protein content by 3.53–6.64%. Conversely, knockout of *GmNFYA14* increased total fatty acid content by 13.51–13.90% and triacylglycerol content by 15.82%. GmNFYA14 did not occupy the promoters of these lipid genes itself. Instead, it interacted with GmWRI1a and attenuated both the transcriptional activity of GmWRI1a and its binding to the promoters of downstream lipid biosynthetic genes such as *GmPKP-β1* and *GmKAS1*. Consistent with this, transcriptome profiling showed that GmNFYA14 suppresses the expression of GmWRI1a-regulated lipid-related genes, restricting seed lipid accumulation.

**Discussion:**

GmNFYA14 is a negative regulator of soybean oil accumulation that acts by dampening GmWRI1a activity rather than by binding target promoters directly. The *GmNFYA14-GmWRI1a* module adds a layer of control to fatty acid synthesis in seeds and provides candidate targets for molecular breeding to improve soybean oil content and agronomic traits.

## 1 Introduction

Soybean (*Glycine max* [L.] Merr.) ranks among the most essential global food crops (Hu et al., 2024), accounting for more than half of the world’s oilseed production and more than a quarter of the protein used globally for both human food and animal feed (Wang et al., 2020). In addition to its nutritional value, soybean oil has attracted widespread attention as a feedstock for biodiesel production (Duan et al., 2023). Relevant projections indicate that global vegetable oil production will need to double over the next 25 years to meet the growing demand for food and biodiesel (Godfray et al., 2010; Foley et al., 2011; Tilman et al., 2011). Therefore, breeding high-quality and high-oil soybean varieties, as well as exploring high-oil-related genes and their molecular mechanisms, are of crucial practical significance (Song et al., 2022). The main component of soybean oil is triacylglycerols (TAGs) (Chapman and Ohlrogge, 2012; Zhang et al., 2019), which include saturated fatty acids (primarily palmitic acid 16:0, followed by stearic acid 18:0) and unsaturated fatty acids (oleic acid 18:1, linoleic acid 18:2, and α-linolenic acid 18:3) (Lee et al., 2007). The biosynthesis of TAGs starts with carbon precursors derived from sucrose, which are first converted into glycolytic intermediates and then transported into plastids for the *de novo* synthesis of fatty acids (Baud, 2018).

Lipid metabolism during soybean seed development is precisely regulated by a variety of transcription factors (Alizadeh et al., 2021), with multiple regulatory modules and signaling pathways collectively involved in modulating this complex physiological process (Wei et al., 2025). Among these regulatory factors, the conserved *LAFL* (*LEC1*, *ABI3*, *FUS3*, and *LEC2*) transcription factor network plays a pivotal role (Chen et al., 2023). By governing hormone synthesis and signaling cascades, it exerts significant effects on amino acid metabolism and triacylglycerol (TAG) synthesis at different stages of soybean seed development (Pelletier et al., 2017; Yang et al., 2025). As a direct downstream target of the *LAFL* network, WRI1 is transcriptionally regulated by three core components (Baud et al., 2007), LEC1, LEC2, and FUS3, forming a classic hierarchical regulatory cascade in the lipid biosynthetic pathway (Manan et al., 2017; Huang et al., 2022; Yang et al., 2022). In soybean, overexpression of *GmWRI1* significantly upregulates the transcription of endoplasmic reticulum-localized TAG synthesis genes such as *PDCT* and *LPAAT*, thereby effectively promoting the accumulation of saturated fatty acid-rich TAGs (Chen et al., 2018, 2020; Guo et al., 2020; Wang et al., 2022). Furthermore, GmWRI1 can bind to AW-box cis-elements or other conserved promoter motifs in target genes to precisely regulate carbohydrate flux and auxin-related signaling pathways (Maeo et al., 2009), enhancing starch degradation, glycolytic metabolism, and *de novo* fatty acid synthesis in developing soybean seeds (Jo et al., 2024). Beyond the LAFL-WRI1 module, numerous other transcription factor families, including *Dof*, *bZIP*, *TZF* (*Tandem CCCH zinc finger*), and *MYB*, also regulate soybean lipid accumulation through independent or synergistic modes (Wang et al., 2007; Song et al., 2013; Liu et al., 2014; Lu et al., 2021).

Nuclear factor Y (NFY), also referred to as CCAAT-binding factor (CBF), functions as a critical transcriptional complex in plants (Mantovani, 1999). NFYA family members are widely implicated in various biological processes, including stress adaptation, nodule development, and overall plant growth (Leyva González et al., 2012; Fornari et al., 2013; Hou et al., 2014; Bu et al., 2020). In soybean, the *NFY* gene family consists of 68 identified members (Quach et al., 2015). However, functional characterization remains limited to only a few soybean *NFYA* genes to date. For instance, the roles of *GmNFYA3*, *GmNFYA5*, and *GmNFYA10* in drought stress responses have been clearly established (Ni et al., 2013; Ma et al., 2020; Yu et al., 2020), while *GmNFYA21* has been verified to modulate flowering time in soybean (Li et al., 2021). GmNFYA11 physically interacts with GmWRI1a, directly triggering *GmOLEO1* expression via CCAAT-box binding and amplifying GmWRI1a’s transactivation capacity on lipid pathway genes to promote seed oil deposition (Gou et al., 2026). GmNFYA boosts seed oil by directly activating GmZF392 and indirectly upregulating *GmZF351*, and the two TZF proteins interact to co-activate lipid biosynthetic genes (Lu et al., 2021). *GmNFYA14* (*SW14*, *Glyma.14G010000*), identified through GWAS and QTL mapping, pleiotropically regulates seed oil and protein contents as well as 100-seed weight by interacting with GmLEC1a/b to disrupt functional NF-Y complexes and repress seed developmental genes (Zhang et al., 2025a). It is also involved in salt stress responses and growth regulation in soybean (Wang et al., 2023a). Collectively, these findings highlight *GmNFYA14* as a promising gene resource for soybean molecular breeding. However, the *GmNFYA14*-associated regulatory network underlying soybean lipid metabolism remains largely unexplored, warranting further investigation.

Herein, we screened for interacting proteins using the master lipid regulatory factor GmWRI1a (Wang et al., 2022) to identify potential genes that may regulate soybean oil content, and *GmNFYA14*, a previously reported lipid regulatory factor, was further investigated to elucidate its regulatory mechanism in soybean lipid metabolism. Our results demonstrated that overexpression of *GmNFYA14* altered fatty acid composition and decreased total fatty acid and triacylglycerol (TAG) accumulation; biochemical experiments revealed that GmNFYA14 interacts with GmWRI1a. Further studies showed that GmNFYA14 inhibits the transcriptional activation activity and DNA-binding ability of GmWRI1a by physically binding to GmWRI1a, without detectably occupying the target promoters itself. RNA-Seq results indicated that the transcriptional levels of downstream lipid synthesis-related genes in *GmNFYA14*-overexpressing soybean were significantly decreased. Collectively, our research clarifies the novel molecular mechanism by which the *GmNFYA14*-*GmWRI1a* module regulates soybean lipid synthesis, laying a foundation for further research on high-oil soybean breeding.

## 2 Materials and methods

### 2.1 Plant materials and cultivation conditions

Soybean cultivar Williams 82 (*Glycine max* (L.) Merr.) was selected as the experimental material in this study. It was utilized for *GmNFYA14* gene cloning, expression profiling analysis, and transgenic transformation experiments. All soybean seedlings were cultivated in a controlled growth chamber, with a diurnal temperature cycle of 26 °C during daytime and 23 °C at night. The cultivation environment was maintained at 70% relative humidity, with a 14 h light and 10 h dark photoperiod.

### 2.2 Yeast two-hybrid and self-activation assay

The coding sequences of *GmNFYA14* and *GmWRI1a* were individuallyinserted into the pGADT7 and pGBT9 vectors, respectively. Because the full-length*GmWRI1a* exhibited strong self-activation when fused to the GAL4 DNA-binding domain (**Supplementary Figure 2**), a C-terminally truncated version, GmWRI1a-NT (amino acids 1–323), which retains both AP2/EREBP domains but lacks the C-terminal transactivation region, was used as the BD bait in Y2H. *GmNFYA14* was expressed as an AD fusion. Full-length GmWRI1a was used in the LCI, BiFC, pull-down, and Co-IP assays, which do not rely on transcriptional activation. Following standard protocols provided by Clontech (Mountain View, CA, USA), the Y2H Gold yeast strain was co-transformed with various recombinant plasmid combinations and cultured on selective media. Empty plasmid combinations (pGADT7 with recombinant pGBT9, and pGBT9 with recombinant pGADT7) were adopted as negative controls. Plasmids pGBT9 (negative control), pGBT9-GmWRI1a and pGBT9-GmWRI1a-NT (lacking the C-terminal fragment) were transformed into yeast strains. Fresh single colonies of each strain were selected and suspended in 0.9% sterile sodium chloride solution, and the OD_600_ value was adjusted to 0.2. Equal volumes of the bacterial suspension were spread on SD/-Trp and SD/-Trp+AbA 800 medium, followed by incubation at 30 °C for 2–4 days. The growth of yeast was observed to identify the self-activation domain of *GmWRI1a*.

### 2.3 Luciferase complementation imaging assay

The coding sequences of *GmNFYA14* and *GmWRI1a* were separately cloned into the pCambia-cLUC and pCambia-nLUC expression vectors, respectively. Subsequently, the two recombinant plasmids, namely cLUC-GmNFYA14 and nLUC-GmWRI1a, were transferred into *Agrobacterium tumefaciens* strain GV3101 through a freeze-thaw transformation method. After activation, the *Agrobacterium* cultures were subjected to centrifugation, and the resulting bacterial pellets were resuspended in sterile distilled water until the optical density at OD_600_ reached 0.3. Various combinations of the recombinant *Agrobacterium* suspensions were then infiltrated into the young leaves of 4-week-old *Nicotiana benthamiana* seedlings. Following a 48-hour incubation period under appropriate conditions, the infiltrated leaf regions were detected and analyzed using a Tanon 5200 imaging system (Tanon, Shanghai, China) to determine the luciferase signal indicative of protein interaction.

### 2.4 Bimolecular fluorescence complementation (BiFC) assay and subcellular localization

The complete coding sequences of *GmNFYA14* and *GmWRI1a* were separately cloned into cYFP-pSUPER and nYFP-pSUPER plasmids to construct fusion expression constructs for BiFC analysis. For subcellular localization investigation, the full-length coding region of *GmNFYA14* with truncated stop codon was inserted into the pBinGFP4 expression vector, yielding the fusion construct 35S::GmNFYA14-eGFP, and the empty 35S::eGFP plasmid was adopted as the negative control. All engineered plasmids were delivered into *Agrobacterium tumefaciens* GV3101 strain through a freeze-thaw transformation method. Bacterial cells after activation were pelleted by centrifugation and reconstituted in infiltration medium to achieve an optical density of 0.3 at 600 nm. The prepared agrobacterial mixtures were separately injected into young foliage of four-week-old *Nicotiana benthamiana* seedlings. After sustained dark incubation for 48 hours, leaf samples were collected for microscopic examination. Yellow fluorescent signals generated from protein interaction and green fluorescent signals reflecting protein distribution were captured and examined by a Zeiss LSM 710 confocal laser scanning microscope (Oberkochen, Germany).

### 2.5 GST Pull-down and coimmunoprecipitation and immunoblotting

The coding sequences of *GmNFYA14* and *GmWRI1a* were inserted into pGEX6p-1 and pET32a vectors separately to generate recombinant constructs, and the corresponding GST-tagged and His-tagged fusion proteins were produced in *Escherichia coli* BL21 cells and subsequently purified. Protein expression was induced with 1 mM isopropyl-β-D-thiogalactopyranoside, where GST-GmNFYA14 was cultured at 16 °C for eight hours and His-GmWRI1a at 37 °C for three hours. Target proteins were isolated at low temperature using GST affinity beads and Ni-NTA 6FF resin kits from respective manufacturers. Afterwards, purified GST-GmNFYA14 and control GST protein were incubated with glutathione beads, while His-tagged GmWRI1a was bound to Ni-NTA resin under identical cooling conditions. After thorough rinsing procedures, all bead-bound samples were processed for subsequent immunoblot detection.

Co-IP assay was performed as described (Wang et al., 2023b). HA-GmNFYA14, GFP, and GFP-GmWRI1a were subsequently introduced into the *A. tumefaciens* strain GV3101 and coinfiltrated into *N. benthamiana* leaves. After 2 d, the crude protein from the treated leaves was extracted with lysis buffer (50 mM Tris-HCl [pH 7.5], 150 mM NaCl, 10 mM EDTA [pH 8.0], 10% glycerol, 1% NP-40, 1 mM DTT, 1 mM PMSF, and 1 × cocktail tablets). The lysates were incubated with agarose beads conjugated with the anti-GFP antibody (KTSM1367, AlpalifeBio) at 4 °C for 3 h. The beads were washed three times with protein lysis buffer. Immunoprecipitates were separated by SDS-PAGE and detected by immunoblotting using anti-HA (30704ES20, Yeasen, 1:5,000 dilution), and anti-GFP (AE012, ABclonal, 1:6,000 dilution) antibodies.

### 2.6 Overexpression and knockout soybean genetic transformation

To generate *GmNFYA14* overexpression lines, the coding sequence of *GmNFYA14* was amplified via PCR and cloned into the pBA002 vector, yielding a *CaMV* 35S promoter-driven *GmNFYA14-*3×*Flag* fusion construct. For targeted knockout of *GmNFYA14*, a specific single guide RNA (sgRNA) targeting the coding exon of *GmNFYA14* was designed using the CRISPR-P 2.0 online tool (http://crispr.hzau.edu.cn/CRISPR2/) and inserted into the CRISPR/Cas9 binary vector pGES201. The selected sgRNA (5’-CACTGAATACCTCGCCACCA-3’, PAM: GGG; on-target score 0.5644) targets exon 1 of *GmNFYA14* (chr14:768,000). Genome-wide off-target prediction by CRISPR-P 2.0 identified 17 candidate off-target sites, of which the highest-scoring site (off-target score 1.000, 0 mismatches, located in *Glyma.02G303800*) was Sanger-sequenced in the homozygous line and showed no editing; all remaining sites contained at least four mismatches with off-target scores ≤ 0.407. Edited individuals were verified by direct Sanger sequencing of the *GmNFYA14* target region, and the evaluated homozygous line carried a 32-bp deletion with no wild-type allele detected. All recombinant plasmids were introduced into soybean cultivar Williams 82 through *Agrobacterium tumefaciens* EHA105-mediated freeze-thaw transformation service provided by BIORUN Biogle GeneTech (Wuhan, Hubei, China). Transgenic positive plants were screened using a glufosinate ammonium resistance selectable marker, and the mutation sites in edited individuals were verified by direct DNA sequencing. The transcript abundance of target genes was quantified using reverse transcription quantitative real-time PCR (RT-qPCR).

### 2.7 RNA extraction and RT-qPCR analysis

Total plant RNA was extracted utilizing the FastPure Universal Plant RNA Extraction Kit from Vazyme Biotech, after which complementary DNA was synthesized with the company’s HiScript II RT reagent kit according to standard protocols. For the tissue-expression assay, leaves, stems, and roots were harvested from 1-week-old seedlings. Quantitative transcriptional analysis was carried out on a QuantStudio™ 5 real-time detection system with SYBR-based qPCR reagents, and the relative expression levels of target genes were calculated via the 2^−ΔΔCT^ algorithm. The *GmActin11* gene was employed as the reference gene for data normalization, and all experimental results were derived from three independent biological replicates to ensure reliability.

### 2.8 Determination of oil, protein, fatty acid and TAG levels

Mature, air-dried seeds (R8 stage, harvested at physiological maturity) of wild-type W82, *GmNFYA14_KO* and *GmNFYA14_OE* plants were used for all oil, protein, fatty acid and TAG measurements. The protein and oil level of soybean samples were measured using an Infratec 1241 near-infrared spectroscopy analyzer manufactured by FOSS. For fatty acid profiling, ground seed samples of 50 milligrams were immersed in cold dichloromethane-methanol solution to perform low-temperature ultrasonic extraction. After solvent separation and nitrogen evaporation, isotopic reference compounds and n-hexane were added to facilitate methyl esterification. Following phase separation, concentrated lipid fractions were redissolved and loaded for gas chromatography-mass spectrometry detection. Analytical separation was carried out on an Agilent DB-23 capillary column with constant helium flow, while the oven temperature was adjusted in a multi-stage gradient program. Mass spectrometry analysis was conducted using an Agilent 5977B instrument with an ionization source, and relevant operational parameters were properly configured. Chromatographic data were processed to extract peak characteristics, and fatty acid contents were quantified through internal standard calibration. All measurement experiments were conducted with three independent biological replicates to ensure data stability and repeatability. Referencing previously established analytical procedures (Xie et al., 2020), triglyceride concentrations in soybean seeds were determined. Seed samples were processed using a mixed methanol-chloroform solvent system supplemented with appropriate internal standards, followed by vigorous shaking for lipid extraction. After aqueous addition and low-speed centrifugation, the organic fraction was separated and concentrated by nitrogen purging. The resulting lipid concentrates were re-dissolved in an equal-volume chloroform-methanol mixture for subsequent measurement, with liquid chromatography and mass spectrometry parameters adopted from the referenced study (Xie et al., 2020).

### 2.9 Dual-luciferase reporter assay

A partial fragment of *GmPKP-β1* with a length of approximately 1500 base pairs was inserted into the pGreenII0800-LUC plasmid, while the regulatory factors *GmWRI1a* and *GmNFYA14* were individually integrated into the pGreenII62-SK expression vector for comparative analysis. These recombinant constructs were delivered into *Agrobacterium tumefaciens* GV3101, and various plasmid combinations were infiltrated into *Nicotiana benthamiana* leaf tissue for subsequent testing. Empty vector constructs were utilized as reference controls to eliminate background interference. For all infiltration combinations the total *Agrobacterium* load was normalized with the empty pGreenII62-SK vector and matched OD_600_, so that the GmWRI1a effector dose was kept constant and differences in LUC activity reflect the presence of specific effectors rather than differences in total effector input. After two days of dark cultivation, luminescence signals were captured using a Tanon 5200 imaging system (Tanon, Shanghai, China). The quantitative measurement and analytical processing of firefly and renilla luciferase activities were carried out in accordance with previously documented methodologies from published literature (Wang et al., 2024).

### 2.10 Electrophoretic mobility shift assay

The coding regions of *GmWRI1a* and *GmNFYA14* were cloned into the pET32a plasmid to facilitate protein expression in *Escherichia coli* BL21 cells. Recombinant His-tagged fusion proteins were generated after induction with 1 mM isopropyl-β-D-thiogalactopyranoside, with 37 °C, 3 h (His-GmWRI1a) and 16 °C, 8 h (His-GmNFYA14) settings for each target protein respectively; subsequent purification was carried out at low temperature using a Ni-NTA affinity resin kit. Multiple biotin-labeled oligonucleotide probes (1-4) containing the conserved AW-box motif were designed from the promoter regions of *GmPKP-β1*, *GmKAS1*, *GmKASIII-2* and *GmACBP1*, and synthesized by Tsingke Biotech (**Supplementary Table S1**). Binding interactions between proteins and target DNA sequences were detected using a EMSA kit (GS009, Beyotime, Nantong, Jiangsu, China), and all binding signals were finally visualized with a Bio-Rad ChemiDoc MP imaging system.

### 2.11 Western blot

Immunoprecipitated samples were separated on 10% NuPAGE^®^ gel and subsequently transferred to polyvinylidene fluoride membranes, which were then treated with 5% skim milk in 1×TBST solution for blocking. After antibody incubation steps, the membranes were rinsed repeatedly with TBST buffer. Target proteins were detected using HRP-labeled His-tag polyclonal antibody and GST-tag monoclonal antibody (Yeasen, China), and final signal visualization was performed with enhanced chemiluminescence reagents in accordance with standard operational protocols.

### 2.12 Transcriptomic analysis

Total RNA was isolated from immature seeds of *GmNFYA14_OE48* and W82 plants at three weeks after flowering (corresponding to approximately the R4-R5 stage of early seed filling), with three biological replicates per genotype, using TRIzol reagent (Thermo Fisher Scientific, USA) according to the manufacturer’s instructions. RNA concentration and purity were determined using a NanoDrop ND-1000 spectrophotometer (Thermo Scientific, USA), and RNA integrity was assessed with an Agilent Bioanalyzer 2100 (Agilent Technologies, USA); all samples fulfilled the criteria of RNA concentration >50 ng/μL, RIN >7.0 and total RNA >1 μg.

Sequencing libraries were constructed from >1 μg of total RNA per sample. Polyadenylated mRNA was enriched with oligo(dT) magnetic beads, fragmented, and converted into strand-specific cDNA by the dUTP method (fr-firstrand); library fragments of ~300 ± 50 bp were subjected to high-throughput paired-end 150 bp sequencing performed on the Illumina NovaSeq X Plus platform by LC-Bio Technology Co., Ltd. (Hangzhou, China).

Raw sequencing reads were subjected to preprocessing with Cutadapt to remove adapters and low-quality sequences, followed by alignment of the processed reads to the *Glycine max* reference genome (Ensembl Plants release 54) using HISAT2 (v2.2.1). Subsequently, transcript-level read counts were calculated and normalized (FPKM) with StringTie (v2.1.6), and differentially expressed genes (DEGs) were identified between *GmNFYA14_OE48* and W82 plants using DESeq2 (v1.22.2), with |log2(fold change)| ≥ 1 and an adjusted P value (Benjamini–Hochberg) < 0.05 as thresholds. All analytical procedures were performed with reference (Gou et al., 2026).

### 2.13 Statistical analysis

All seed compositional traits were measured on three biological replicates per genotype (n = 3). The experimental unit was a pooled seed sample: each biological replicate consisted of mature seeds bulked from 3–5 plants of the same genotype grown in the same batch, and individual plants were not treated as replicates. Data are presented as mean ± SD; error bars in all figures denote SD. Pairwise comparisons were conducted using two-tailed Student’s *t*-test (*P < 0.05, **P < 0.01). One-way ANOVA followed by Tukey’s HSD *post hoc* test was used for multi-group comparisons. Means ± SD, replicate numbers, pooling strategy, and statistical tests for all measured traits are provided in **Supplementary Table S4**-**S10**.

## 3 Results

### 3.1 Physical interaction between GmNFYA14 and GmWRI1a

GmWRI1a functions as a key regulator of soybean oil biosynthesis. To further elucidate theunderlying molecular mechanisms, a screen for potential interacting partners of GmWRI1a was conducted, leading to the identification of *GmNFYA14* (*Glyma.14G010000*), a member of the Nuclear Factor Y (NFY) transcription factor family that is highly expressed in seeds (**Supplementary Figures 1A, B**) as a GmWRI1a-binding protein. Because full-length GmWRI1a autoactivated the yeast reporterswhen fused to the GAL4 DNA-binding domain, we generated a C-terminally truncated bait, GmWRI1a-NT(aa 1–323), which retains both AP2 domains but lacks the C-terminal transactivation domain and showed no self-activation (**Supplementary Figure 2**). Yeast cells co-expressing BD-GmWRI1a-NT and AD-GmNFYA14 grew on quadruple-dropout medium, indicating that GmWRI1a interacts with GmNFYA14 in yeast cells (**Figure 1A**). To corroborate this interaction in planta and determine the subcellular compartment in which it occurs, luciferase complementation imaging (LCI) and bimolecular fluorescence complementation (BiFC) assays were carried out in *Nicotiana benthamiana* leaf cells. Co-expression of nLUC-GmWRI1a and cLUC-GmNFYA14 produced a detectable luminescent signal, confirming that GmWRI1a interacts with GmNFYA14 in plant cells (**Figure 1B**). Consistently, BiFC assays revealed yellow fluorescent protein (YFP) signals confined to the cell nucleus, which co-localized with both the nuclear marker mCherry and the subcellular localization pattern of GmNFYA14 (**Figure 1C**; **Supplementary Figure 1C**). In addition, a GST pull-down assay was performed to confirm this interaction *in vitro*. The results demonstrated that His-GmWRI1a specifically bound to GST-GmNFYA14, whereas no interaction was detected between His-GmWRI1a and GST alone (**Figure 1D**). Moreover, Co-IP assays further substantiated the physical association between GmWRI1a and GmNFYA14 in planta (**Figure 1E**). Notably, full-length GmWRI1a was used in all of these assays, as well as in the subsequent EMSA and dual-luciferase experiments. Taken together, these findings provide compelling evidence for a direct physical interaction between GmNFYA14 and GmWRI1a.

**Figure 1:**
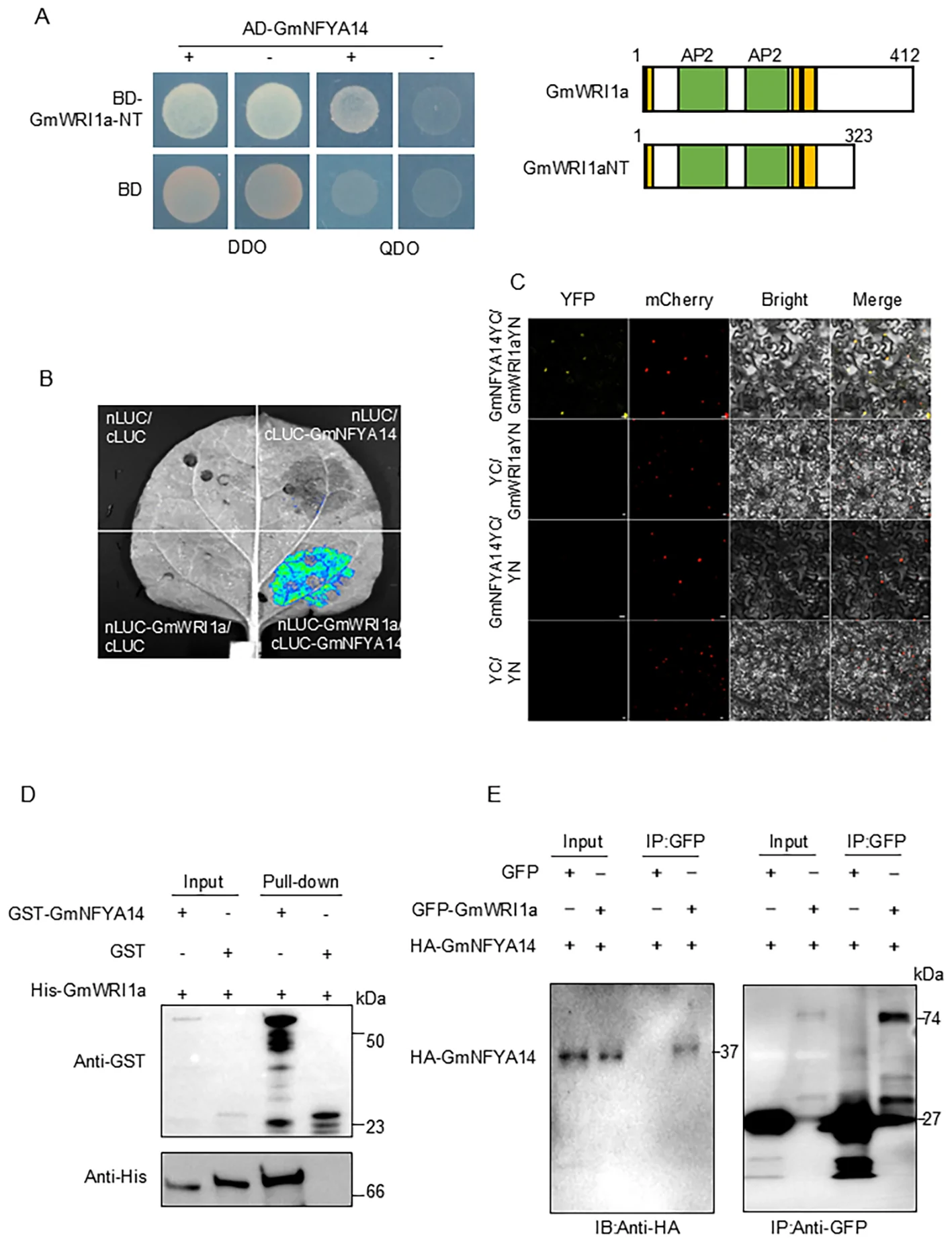
GmNFYA14 forms a physical interaction with GmWRI1a. **(A)** Y2H assays were carried out to verify the interaction between GmWRI1a and GmNFYA14. Transformed yeast strains were cultivated on DDO (SD−Leu/Trp) and QDO (SD−Ade/His/Leu/Trp) selective culture media. The combination of BD-GmWRI1a-NT (pGBT9-GmWRI1a-NT) and empty AD vector (pGADT7) was applied to detect self-activation, while two experimental groups, including empty BD (pGBT9) paired with empty AD (pGADT7) and empty BD combined with AD-GmNFYA14 (pGADT7-GmNFYA14), served as negative control groups. The right panel illustrates the GmWRI1a protein structure. Yellow boxes denote low compositional complexity regions, and green boxes indicate AP2 domains. **(B)** LCI assays were utilized to verify the interaction between GmWRI1a and GmNFYA14 in *Nicotiana benthamiana* leaves. The GmWRI1a protein was fused to the N-terminal fragment of luciferase to construct the nLUC-GmWRI1a fusion protein, while GmNFYA14 was linked with the C-terminal luciferase fragment to generate cLUC-GmNFYA14. The co-expression of nLUC-GmWRI1a and cLUC-GmNFYA14 was set as the experimental group, whereas three combinations, including nLUC-GmWRI1a with empty cLUC, empty nLUC with cLUC-GmNFYA14, and dual empty vectors of nLUC and cLUC, were adopted as control groups. **(C)** BiFC experiments were performed to investigate the interaction between GmWRI1a and GmNFYA14 in *Nicotiana benthamiana* leaves. GmWRI1a was fused to the N-terminal fragment of EYFP to produce the GmWRI1a-YN construct, while GmNFYA14 was linked to the C-terminal EYFP segment to generate GmNFYA14-YC. Three independent combinations, namely GmWRI1a-YN with empty YC, empty YN with GmNFYA14-YC, and blank YN-YC vectors, were utilized as negative controls. Meanwhile, the p35S::NLS-mCherry reporter was applied to mark nuclear localization. Bar = 20 µm. **(D)** *In-vitro* GST pull-down experiments were conducted to analyze the interaction between GST-tagged GmNFYA14 and His-tagged GmWRI1a via GST affinity beads. The input sample was used as the positive control for the assay. **(E)** GmNFYA14 interacts with GmWRI1a in the Co-IP assays. The constructs HA-GmNFYA14 and GFP-GmWRI1a were cotransfected into *N. benthamiana* leaves. Crude proteins were extracted and incubated with GFP beads. HA-GmNFYA14 protein in the immunoprecipitated fraction was detected by the anti-HA antibody. Three independent experiments were performed; representative images are presented here.

### 3.2 Functional role of GmNFYA14 in fatty acid and triacylglycerol biosynthesis in soybean

*GmNFYA14* knockout mutants (*GmNFYA14_KO*) were generated by CRISPR/Cas9-mediated gene editing. One independent *GmNFYA14_KO* soybean line harbored a 32-base-pair deletion within the target gene, resulting in a corresponding alteration in the GmNFYA14 protein sequence (**Figure 2A**). Further screening of the transformed population yielded no additional edited allele, so all knockout data reported here derive from this single editing event. Two observations nevertheless support the phenotype. Independent *sw14* alleles in the same W82 background show the same increase in seed oil accompanied by reduced protein content (Zhang et al., 2025a), and the opposite manipulation produces the opposite result: three independent *GmNFYA14*-overexpressing lines all showed reduced oil and increased protein contents (**Figures 3B, C**), and two of them showed reduced total FA and TAG contents (**Figures 3E, F**). The reciprocal phenotypes of the knockout and overexpression lines, together with the independently published *sw14* alleles, strongly support the proposed role of *GmNFYA14* in seed oil accumulation. The two sets of transgenic lines were produced in separate transformation events and their phenotypes move in opposite directions; a problem caused by the transformation procedure itself would be expected to push both sets the same way. Because only one knockout event was recovered, however, a contribution from line-specific background effects cannot be formally excluded, and additional independent alleles or a complementation test would be needed to settle this. Subsequently, systematic analyses of fatty acid (FA) composition and triacylglycerol (TAG) accumulation were performed. The total FA content in *GmNFYA14_KO* seeds increased significantly by 13.51%–13.90% relative to that of W82 rising from 104.12 to 118.33 mg/g dry weight (DW) (**Figure 2B**). Likewise, TAG content was significantly elevated by 15.82% (from 73.25 to 84.84 mg/g) in *GmNFYA14_KO* seeds compared with W82 (**Figure 2C**). These findings are consistent with the previously reported negative regulatory role of *GmNFYA14* in oil accumulation (Zhang et al., 2025a). Field evidence from an independent study provides further information on the agronomic effects of *GmNFYA14* disruption. Zhang et al. reported that *sw14* mutants, which carry mutations in the same gene and were generated in the W82 background, exhibited increased seed oil content together with reduced 100-seed weight and protein content. In contrast, no significant differences were observed in plant height, node number, branch number, or seed number per plant compared with the wild type under field conditions at two latitudes (Zhang et al., 2025a). These findings suggest that loss of *GmNFYA14* does not markedly affect the agronomic traits examined, despite its effects on seed composition and seed weight. However, the present study was conducted under controlled growth-chamber conditions and did not include field trials. Therefore, the field performance and agronomic effects of our *GmNFYA14_KO* mutant remain to be evaluated in future studies.

**Figure 2:**
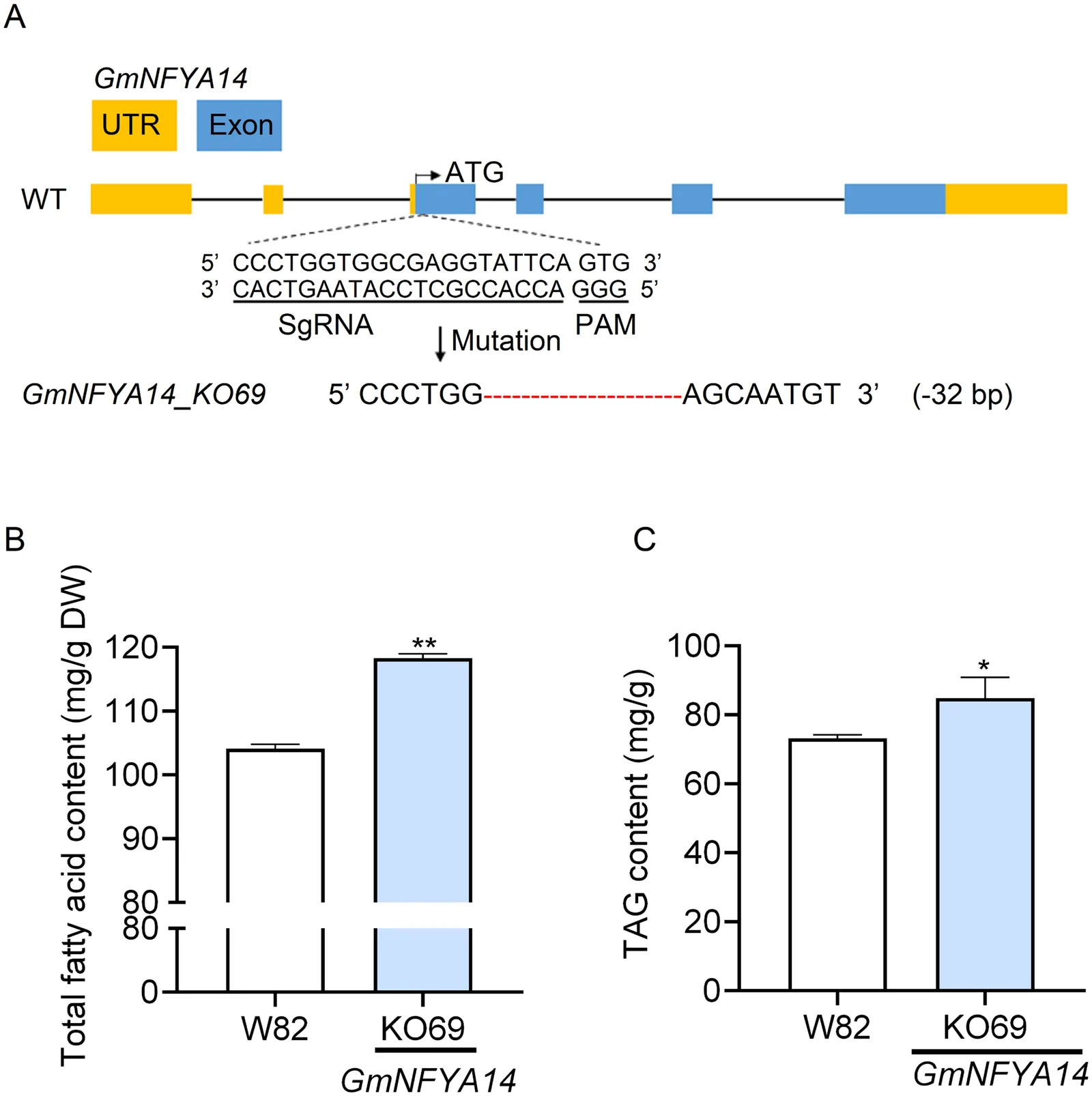
Knockout of *GmNFYA14* elevates the content of total fatty acids and triacylglycerol in soybean seeds. **(A)** Schematic illustration of CRISPR-Cas9-induced mutagenesis of *GmNFYA14*. The sgRNA (5’-CACTGAATACCTCGCCACCA-3’) targets the first exon of *GmNFYA14*. Red dashed lines indicate the 32-bp deletion identified in the homozygous line, which causes a frameshift and a premature stop codon, resulting in a truncated protein. **(B, C)** Total fatty acid and TAG contents were compared between seeds of wild-type W82 and *GmNFYA14* knockout line (KO69). All measured data are expressed as mean ± standard deviation (SD) from three biological replicates, each a pooled seed sample from 3–5 plants of the same genotype. Statistical significance was evaluated by Student’s *t*-test, with asterisks indicating significant differences (*P < 0.05, **P < 0.01).

**Figure 3:**
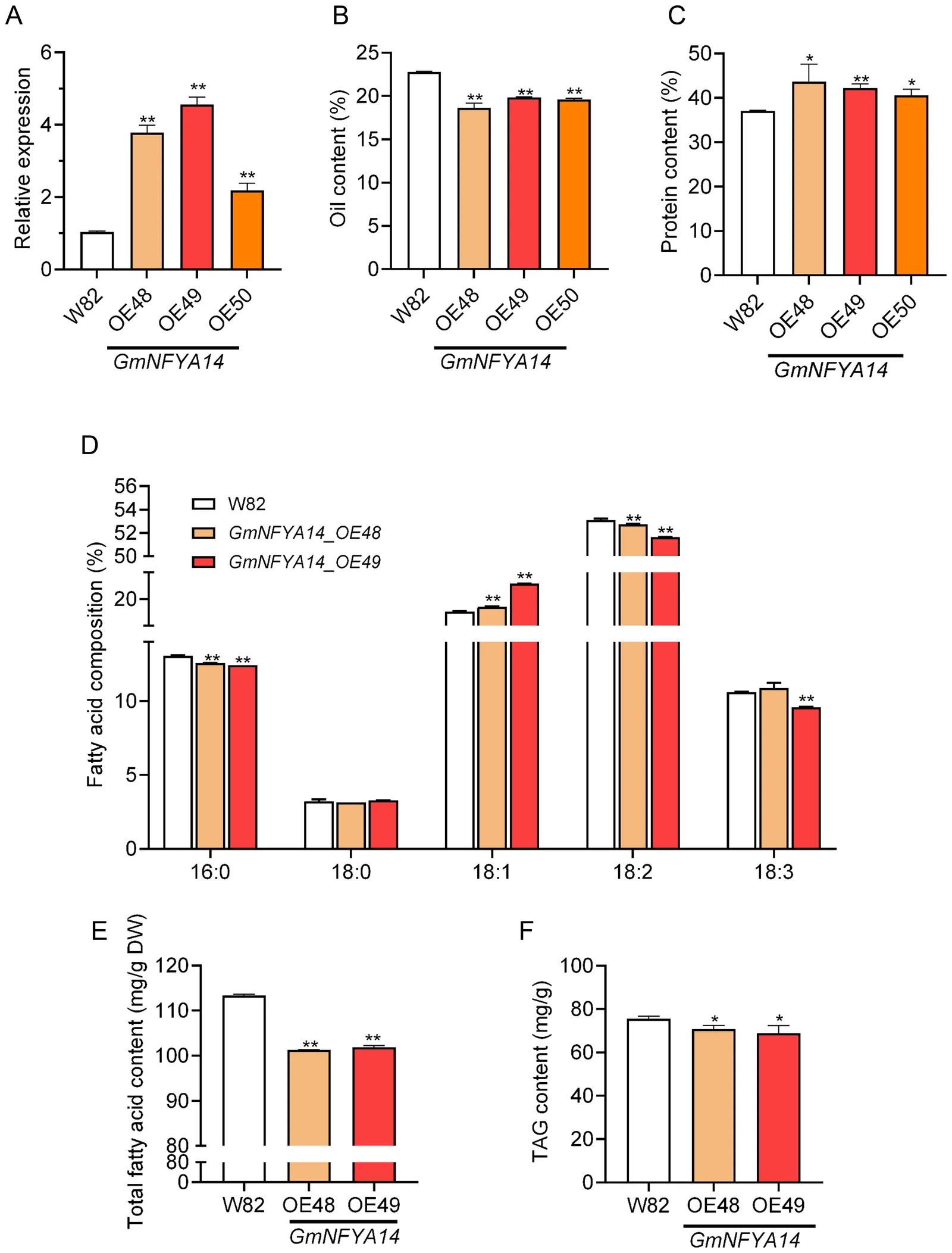
Overexpression of *GmNFYA14* decreases fatty acid, triacylglycerol and lipid accumulation in soybean seeds. **(A)** The expression level of *GmNFYA14* was analyzed in seed tissues of wild-type W82 and *GmNFYA14_OE* soybean lines. Seed samples were collected at 21 days after flowering (DAF), with OE48, OE49 and OE50 representing three independent transgenic OE lines. *GmActin11* was used as the reference gene for expression analysis. **(B, C)** Seed oil and protein contents of W82 and *GmNFYA14_OE* plants were measured. **(D–F)** Variations in fatty acid composition, total fatty acid and triacylglycerol (TAG) content were compared between W82 and *GmNFYA14_OE* seeds. Fatty acid components include palmitic acid (16:0), stearic acid (18:0), oleic acid (18:1), linoleic acid (18:2), and α-linolenic acid (18:3). Measured data are summarized as mean ± SD from three biological replicates, each a pooled seed sample from 3–5 plants of the same genotype, and significant differences were assessed using Student’s *t*-test, with asterisks denoting statistical differences (*P < 0.05, **P < 0.01).

To further elucidate the molecular mechanism underlying *GmNFYA14*-mediated regulation of soybean lipid biosynthesis, *GmNFYA14*-overexpressing transgenic soybean lines (*GmNFYA14_OE*) were generated via *Agrobacterium tumefaciens*-mediated cotyledonary node transformation using W82 as the recipient cultivar (**Figure 3A**; **Supplementary Figure 3**). Seed oil content was significantly reduced in *GmNFYA14_OE* lines compared with W82, with decreases ranging from 2.96% to 4.13%, corresponding to relative reductions of 13.00%-18.14% (**Figure 3B**). Conversely, seed protein content was significantly elevated in *GmNFYA14_OE* lines, with increases of 3.53%–6.64%, corresponding to relative increases of 9.52%-17.92%, relative to W82 (**Figure 3C**). Two representative overexpression lines (OE48 and OE49) with consistent transgene expression across multiple batches were selected for subsequent fatty acid and TAG analyses. Total FA content in *GmNFYA14_OE* seeds also decreased markedly by 9.90%–10.78% compared with W82 (**Figure 3E**). TAG content in *GmNFYA14_OE* seeds was reduced by 6.32%–8.88% relative to W82 (**Figure 3F**). Regarding fatty acid composition, the contents of linoleic acid (18:2), palmitic acid (16:0) were substantially lower in *GmNFYA14_OE48* and *GmNFYA14_OE49* lines than in W82, decreasing by 0.71%-2.76% (18:2) and 3.77%-4.69% (16:0), respectively, whereas oleic acid (18:1) content was significantly higher increasing by 2.97%-16.85% (**Figure 3D**). These results collectively demonstrate that *GmNFYA14* overexpression not only alters fatty acid composition but also reduces soybean seed oil and total FA accumulation while enhancing seed protein content, thereby confirming the negative role of *GmNFYA14* in soybean oil biosynthesis.

### 3.3 GmNFYA14 acts as an inhibitor to repress the transcriptional activation activity of GmWRI1a

To further investigate the molecular mechanism underlying the cooperative regulation of oil content by *GmNFYA14* and *GmWRI1a*, luciferase (LUC) activation assays were performed in *Nicotiana benthamiana* leaves. Based on the established regulatory role of GmWRI1a in activating its target gene *GmPKP-β1* (plastidial pyruvate kinase β subunit 1, a key gene involved in lipid biosynthesis) (Jo et al., 2024; Gou et al., 2026), LUC fluorescence intensity and enzyme activity driven by the *GmPKP-β1* promoter (pro*GmPKP-β1*) were measured to evaluate the effect of GmNFYA14 on the transcriptional activation function of GmWRI1a (**Figure 4A**). Transient expression of *GmWRI1a* significantly enhanced LUC activity relative to the empty vector, whereas expression of *GmNFYA14* alone did not affect pro*GmPKP-β1* activity, indicating that GmNFYA14 neither activates this promoter nor represses it as a standalone DNA-bound factor under these assay conditions. By contrast, co-expression of *GmNFYA14* and *GmWRI1a* markedly reduced LUC activity compared with alone, demonstrating that GmNFYA14 suppresses the transcriptional activation of *GmPKP-β1* mediated by GmWRI1a (**Figures 4B, C**). Because this reduction was strictly dependent on the presence of GmWRI1a and was not accompanied by any autonomous effect of GmNFYA14 on the promoter, the inhibition is exerted on GmWRI1a rather than at the level of the promoter.

**Figure 4:**
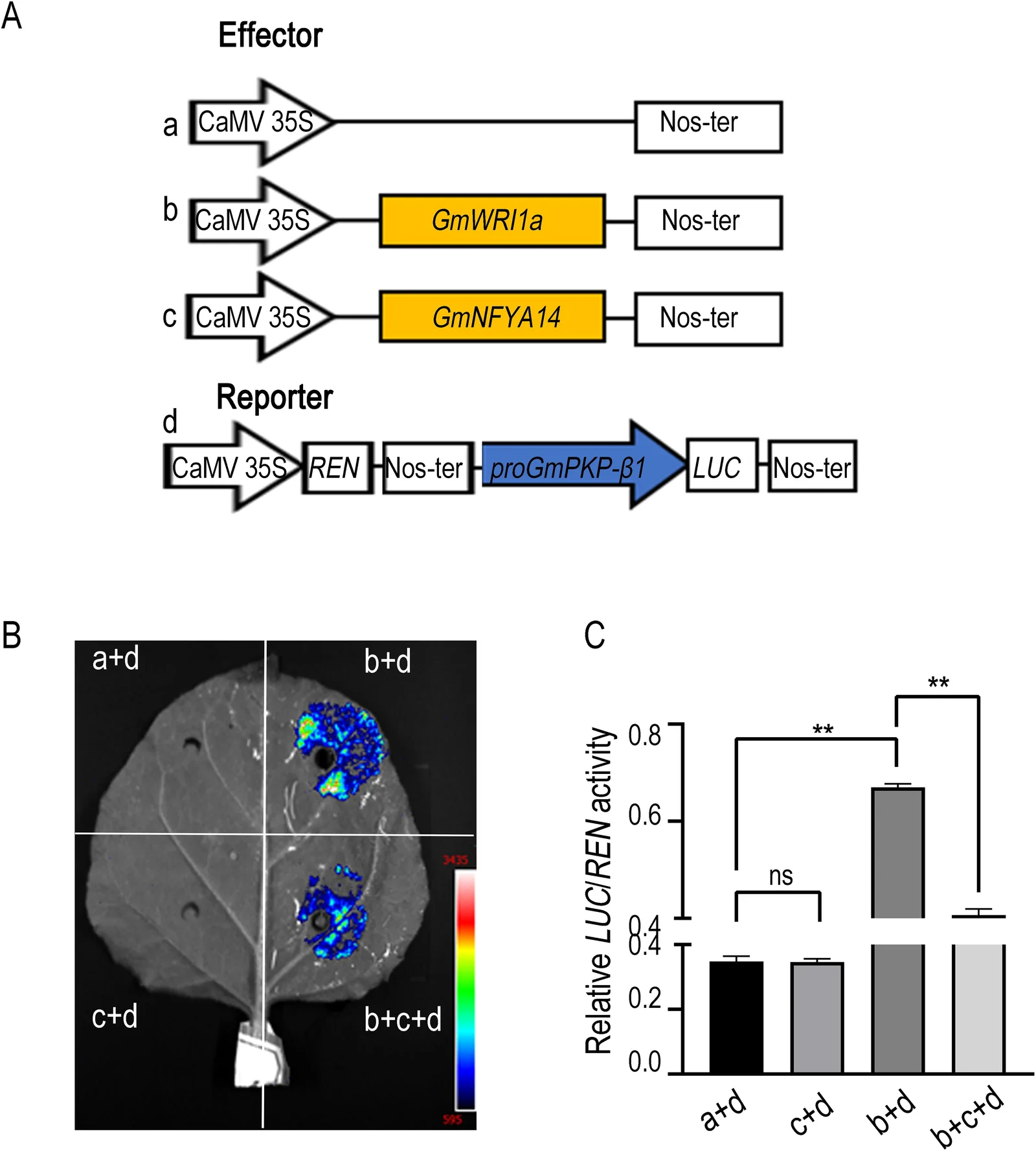
GmNFYA14 represses the transcriptional activity of GmWRI1a. **(A)** Schematic illustration of effector and reporter constructs used in this study. The effector vectors pGreenII62-SK-GmWRI1a and pGreenII62-SK-GmNFYA14 were constructed, while pGreenII0800-LUC-proGmPKP-β1 served as the reporter plasmid. The empty pGreenII62-*SK* vector was applied as the negative control. **(B)** Luciferase activity detection under different effector-reporter combination treatments. The gradual color transition from red to blue represents a gradual reduction in LUC activity levels. **(C)** LUC/REN contributed to the detection of transactivation activity. Data are presented as means ± SD, with each value derived from three biological replicates. One-way ANOVA revealed a highly significant difference among the four groups. Tukey’s HSD *post hoc* test showed that the mean value in the b+d group was significantly higher than in all other groups (*P* < 0.01, **). The b+c+d group was significantly higher than both a+d and c+d groups (*P* < 0.01, **). No significant difference was observed between the a+d and c+d groups.

### 3.4 GmNFYA14 represses the DNA-binding capacity of GmWRI1a

Furthermore, an EMSA was performed to evaluate the effect of GmNFYA14 on the DNA-binding activity of GmWRI1a. GmWRI1a bound specifically to probes 1–4, which contain the AW-box from the promoter regions of *GmPKP-β1* (plastidial pyruvate kinase β subunit 1), *GmKAS1* (β-ketoacyl-ACP synthase I), *GmKASIII-2* (3-ketoacyl-ACP synthase III), and *GmACBP1* (acyl-coenzyme A-binding protein 1), respectively. The addition of unlabeled competitive probes attenuated the binding signal, whereas the inclusion of GmNFYA14 protein diminished the binding affinity of GmWRI1a for the labeled probes (**Figure 5**; **Supplementary Figure 4**). In EMSA with purified recombinant proteins, His-GmNFYA14 alone did not bind the probes, and its attenuation of the GmWRI1a–DNA signal without generating a slower-migrating complex indicates that GmNFYA14 directly reduces the DNA-binding capacity of GmWRI1a via binary interaction, rather than assembling a stable repressive complex at the AW-box. Thus, the attenuation of DNA binding was observed for AW-box elements derived from four independent GmWRI1a target genes, indicating that this mode of action is not restricted to an individual promoter. Importantly, the attenuation of GmWRI1a DNA-binding activity by GmNFYA14 was reproduced in a cell-free system containing only purified recombinant proteins, in the absence of chromatin, histone-modifying enzymes, or plant-specific corepressors. These results therefore support a model in which GmNFYA14 directly interferes with the GmWRI1a–DNA interaction rather than acting primarily through chromatin-mediated transcriptional repression. Collectively, these findings demonstrate that GmNFYA14 inhibits fatty acid and lipid accumulation in soybean by attenuating both the transcriptional activation and DNA-binding activities of GmWRI1a.

**Figure 5:**
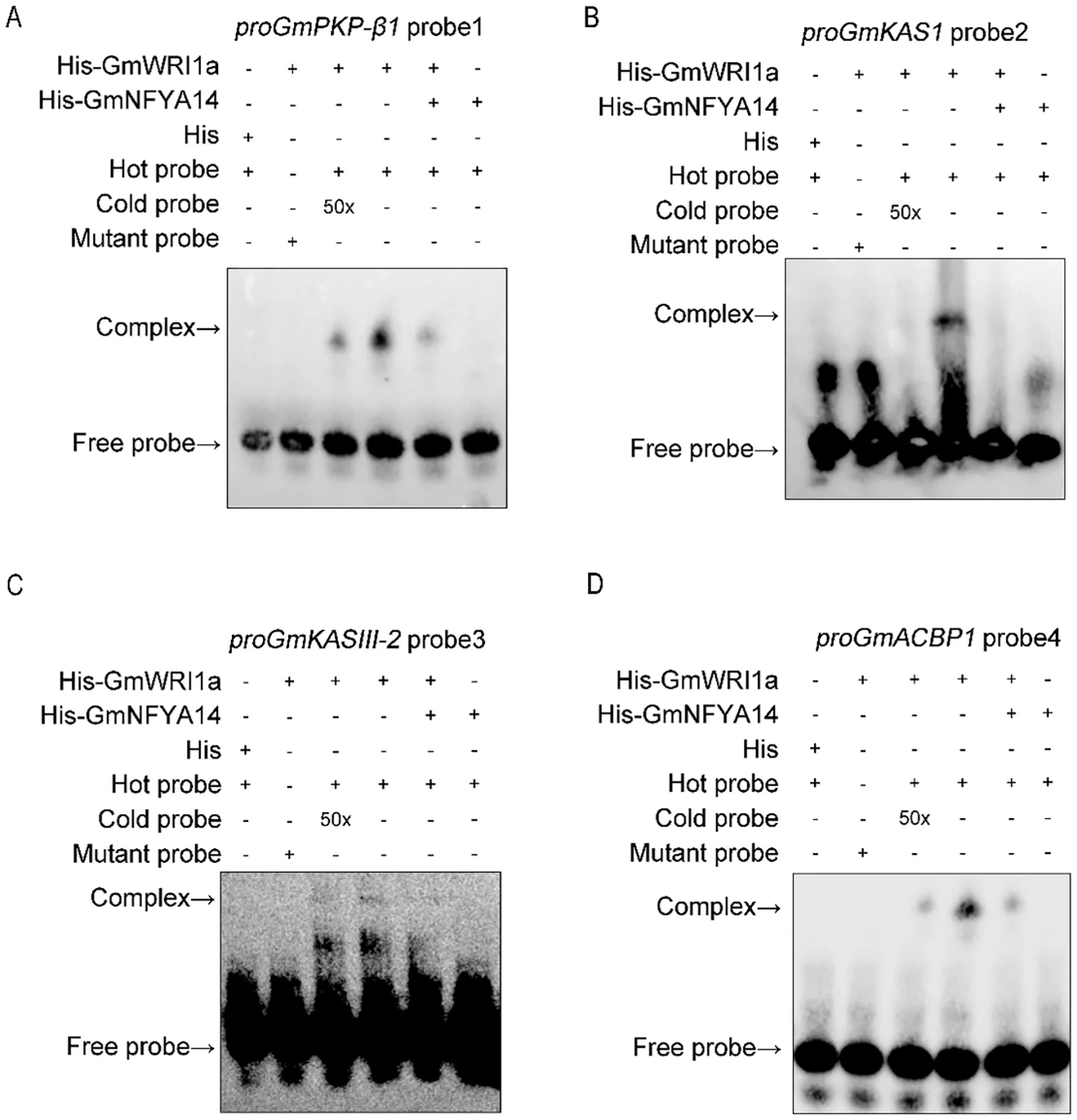
GmNFYA14 weakens the DNA-binding capacity of GmWRI1a to AW-box-containing promoters. EMSA showing that GmWRI1a bound to the AW-box in the *proGmPKP-β1* **(A)**, *proGmKAS1* **(B)**, *proGmKASIII-2* **(C)** and *proGmACBP1* **(D)**, and GmNFYA14 decreased the binding ability. His-GmNFYA14 alone did not bind to the probes (lane 6). Non-labeled competitive probes and motif-deficient mutant probes were further applied to validate the binding specificity.

### 3.5 GmNFYA14 modulates gene expression in response to oil biosynthesis

To elucidate the molecular regulatory mechanisms underlying *GmNFYA14*-mediated seed oil biosynthesis in soybean, transcriptome sequencing was performed on seeds at 21 days after flowering (DAF) from *GmNFYA14_OE48* and the control W82 (**Supplementary Table S2**-**S3**). At 21 DAF, soybean seeds are in the mid-seed-filling stage, a period of rapid oil accumulation, when genes involved in FA biosynthesis display near-maximal transcriptional activity that peaks at approximately 20 DAF and declines thereafter (Yang et al., 2019). Comparative transcriptomic profiling identified 4,418 significantly up-regulated and 5,933 significantly down-regulated genes in *GmNFYA14_OE48* relative to W82 (**Figure 6A**). These differentially expressed genes (DEGs) represent candidate targets of *GmNFYA14* and may participate in the regulation of soybean seed oil content. Kyoto Encyclopedia of Genes and Genomes (KEGG) enrichment analysis revealed that multiple pathways, including those associated with lipid metabolism, were significantly enriched (**Figure 6B**). These findings underscore the intimate association between *GmNFYA14* and seed lipid anabolism in soybean. The majority of genes involved in FA biosynthesis and TAG synthesis (Jo et al., 2024) were downregulated in *GmNFYA14_OE48* compared with W82 (**Figure 6C**). Notably, all four GmWRI1a target genes (*GmPKP-β1*, *GmKAS1*, *GmKASIII-2*, and *GmACBP1*) (Jo et al., 2024) examined by EMSA were also downregulated at the transcript level in *GmNFYA14_OE48*, and the reliability of these changes was supported by an independent overexpression line (**Figures 6C, D**). We further validated a subset of key differentially expressed genes in the independentOE49 line by qPCR, and most of these expression alterations were reproduced, reinforcing thereliability of the observed transcriptomic responses (**Supplementary Figure 5**). Collectively, these findings demonstrate that *GmNFYA14* acts as a negative regulator through repression of *GmWRI1a*-modulated lipid biosynthetic genes, thereby attenuating fatty acid and oil accumulation in soybean seeds.

**Figure 6:**
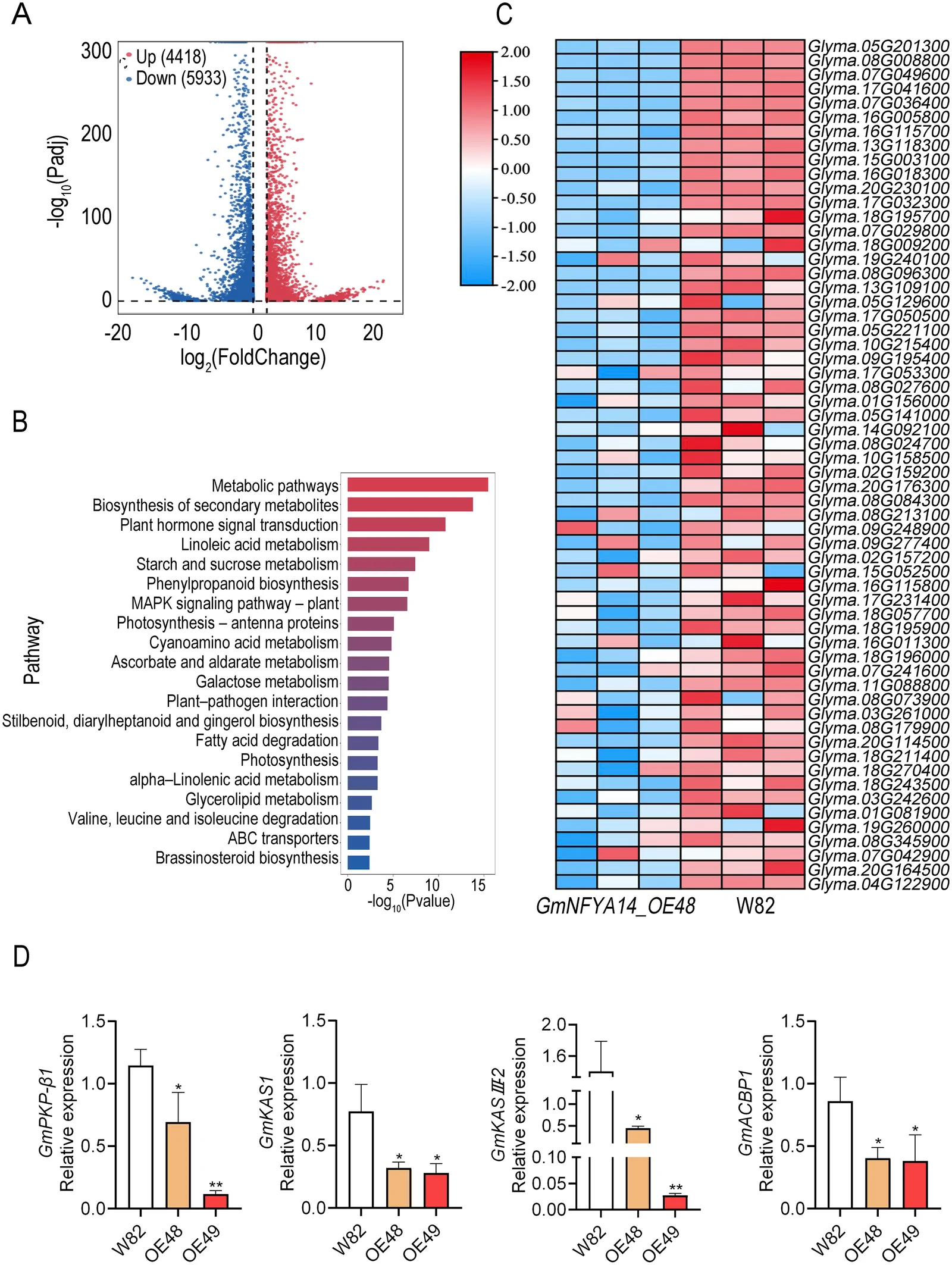
Transcriptome analysis of *GmNFYA14_OE48* and W82 seeds. **(A)** Volcano plot illustrating differentially expressed genes (DEGs) between *GmNFYA14_OE48* and W82 seeds. **(B)** KEGG pathway enrichment analysis of DEGs between *GmNFYA14_OE48* and W82 seeds. **(C)** Based on transcriptome sequencing data of *GmNFYA14_OE48* and W82 seeds, an FPKM expression heatmap was constructed for genes involved in fatty acid biosynthesis and TAG synthesis. All heatmap data were derived from three biological replicates. **(D)** RT-qPCR was performed to detect the transcript levels of *GmWRI1a* target genes (*GmPKP-β1*, plastidial pyruvate kinase β subunit 1; *GmKAS1*, β-ketoacyl-ACP synthase I; *GmKASIII-2*, 3-ketoacyl-ACP synthase II*I*; *GmACBP1*, acyl-coenzyme A-binding protein 1) in seeds of wild-type W82 and *GmNFYA14*-overexpressing line OE48 and OE49; data are presented as mean ± SD from three biological replicates (n = 3); error bars denote SD.

## 4 Discussion

### 4.1 The multiple regulatory mode and network interaction of GmNFYA14 in lipid biosynthesis

Previous studies have shown that GmNFYA14 physically interacts with GmLEC1a/b and disrupts the assembly of the non-canonical NFY complex consisting of GmLEC1, GmNFYC2 and GmbZIP67 (Zhang et al., 2017; Jo et al., 2019). This interaction suppresses GmLEC1-dependent transcriptional activation during seed development and downregulates fatty acid biosynthetic genes (Yamamoto et al., 2009; Mendes et al., 2013; Zhang et al., 2025a), which is consistent with our findings regarding the inhibitory role of *GmNFYA14* in lipid biosynthesis. Notably, this study expands this regulatory network by identifying a novel pathway centered on GmWRI1a. GmNFYA14 directly interacts with GmWRI1a (**Figure 1**) and represses its transcriptional activity toward *GmPKP-β1* and other genes involved in FA and TAG biosynthesis (**Figures 6C, D**). By interfering with both the LEC1-mediated seed maturation pathway and the GmWRI1a-driven lipid synthesis pathway, this dual-targeting mechanism clarifies the integrated function of GmNFYA14 in modulating oil accumulation in soybean seeds. In this study, both *in-vitro* GST pull-down and *in-vivo* Co-IP assays and other assays confirm the physical interaction between GmNFYA14 and GmWRI1a. GmNFYA14 sequesters GmWRI1a from its targets by reducing its DNA-binding capacity; it alone binds neither AW-box probes (**Figure 5**) nor *proGmPKP-β1* (**Figure 4**), and therefore acts as an inhibitor of transcription factor assembly rather than a DNA-bound repressor. Since *GmWRI1a* is an AP2/ERF factor unrelated to NF-YB/NF-YC, and the GmNFYA14-mediated inhibition of GmWRI1a was reconstituted with purified proteins, the *GmNFYA14*–*GmWRI1a* module is biochemically separable from NF-Y trimer formation. Moreover, because GmWRI1a acts downstream of GmLEC1 in the LAFL cascade (Kong et al., 2020), the simultaneous repression of both the upstream GmLEC1-containing activator complex (Zhang et al., 2025a) and the downstream effector GmWRI1a (this study) by GmNFYA14 constitutes a coherent dual-node inhibition of seed lipid biosynthesis; whether the two branches compete for overlapping surfaces on GmNFYA14 remains to be tested.

Transcriptional repression may occur through direct promoter-associated mechanisms or through interference with a transcriptional activator. Our results support the latter model for GmNFYA14. GmNFYA14 alone did not bind the AW-box probes or alter *proGmPKP-β1* activity, whereas repression was observed only in the presence of GmWRI1a (**Figures 4**, **5**); a repressor acting at these promoters would be expected to change their activity on its own. Moreover, GmNFYA14 reduced GmWRI1a-DNA binding in a cell-free system containing only purified recombinant proteins, reproducing the effect seen in planta and indicating that this inhibition requires neither chromatin nor plant-specific corepressors. The absence of an additional slower-migrating complex in EMSA further suggests that GmNFYA14 does not form a stable ternary complex with GmWRI1a on DNA. The same attenuation was seen for AW-box elements from four independent GmWRI1a target genes, which is difficult to reconcile with locus-specific recruitment of a corepressor. The GmWRI1a region that interacts with GmNFYA14 contains both AP2 DNA-binding domains (**Figure 1A**; **Supplementary Figure 2**), supporting a model in which GmNFYA14 interferes with GmWRI1a binding to DNA. Whether this reflects steric hindrance of the AP2 domains or an interaction-induced conformational change remains unresolved, and separating the two will require deletion mapping of GmWRI1a. Competitive inhibition likewise cannot be concluded here, because only a single effector ratio was tested; dose-dependent dual-luciferase titration and complementary EMSA titration would settle this, and since such measurements were not made we refrain from describing the inhibition as competitive. Thus, at the promoters examined here, GmNFYA14 suppresses GmWRI1a activity through direct protein-protein contact rather than through promoter-bound repression. Neither of these open points affects the conclusions above, which rest only on the requirement for direct physical contact between the two proteins.

Nevertheless, we cannot rule out that additional cellular co-factors reinforce the GmNFYA14-dependent repression of GmWRI1a in developing soybean seeds. Further domain-truncation and point-mutation experiments are required to dissect the critical amino-acid residues responsible for protein interaction and functional repression. Loss of both interaction and repressive function upon GmNFYA14 mutation would indicate that direct physical binding is the primary driver of GmWRI1a inhibition. Such mechanistic dissection will be pursued in our future work. *GmNFYA14*-overexpressing seeds exhibited higher protein levels than WT plants (**Figure 3C**), whereas *GmNFYA14* knockout (*GmNFYA14_KO*) lines showed the opposite phenotype (**Figure 2**). GmNFYA14 may sequester GmLEC1a/b to indirectly repress storage protein genes, as GmLEC1a/b are well-characterized activators of glycinin and β-conglycinin (Zhang et al., 2017; Jo et al., 2019). It also redirects metabolic flux from FA and TAG production to amino acid and storage protein synthesis, a pattern supported by the inverse relationship between oil and protein contents observed in natural soybean accessions carrying the *SW14* locus (Zhang et al., 2025a). In addition, GmNFYA14 may regulate storage protein gene expression by directly recognizing CCAAT motifs (Laloum et al., 2013), although this regulatory mode requires further experimental verification. ChIP-based promoter-occupancy data were not obtained in this study, and we cannot formally exclude that GmNFYA14 also associates with CCAAT-box elements of some target promoters through NF-YB/NF-YC partners, independently of GmWRI1a. For the promoters examined here (*GmPKP-β1*, *GmKAS1*, *GmKASIII-2*, and *GmACBP1*), however, this is not supported, as GmNFYA14 alone neither bound the AW-box probes (**Figure 5**) nor affected *proGmPKP-β1* activity in the presence of endogenous NF-YB/NF-YC (**Figure 4**). ChIP-based analyses will be required to resolve this point. Although RNA-seq data were obtained from a single transgenic line, qPCR validation in an independent transgenic line confirmed a number of key expression changes, suggesting that these transcriptional reprogramming events are largely attributable to *GmNFYA14* overexpression. Further investigations will be required to fully dissect the regulatory network downstream of *GmNFYA14*. In summary, *GmNFYA14* orchestrates a complex regulatory network by disturbing core transcription complexes, impairing key transcription factor activities, and possibly binding directly to downstream target genes, thereby comprehensively controlling lipid biosynthesis and oil deposition in soybean seeds.

### 4.2 A bidirectional regulatory module of GmNFYA11/GmNFYA14 modulating oil accumulation in soybean

The specific interaction between transcription factors is a core molecular event that regulatesthe sophisticated network of plant lipid metabolism (Boulardet al., 2018; Wei et al., 2025). Combined with previous work, this study shows that two NFYA family members, GmNFYA11 and GmNFYA14, directly interact with GmWRI1a, a key regulator of soybean lipid metabolism. Although a C-terminally truncated GmWRI1a (GmWRI1a-NT, aa 1–323) was used as bait in Y2H because the full-length protein autoactivates the yeast reporters through its C-terminal transactivation domain (**Supplementary Figure 2**), this does not affect our conclusions: GmWRI1a-NT retained the interaction in yeast, and the full-length GmWRI1a interacted with GmNFYA14 in four transcription-independent assays (LCI, BiFC, pull-down, and Co-IP), collectively indicating that the interaction is genuine and does not require the C-terminal region. Moreover, all functional assays were performed with full-length GmWRI1a, and GmNFYA14 repressed both its DNA-binding and transactivation activities, indicating that the interaction is operative on the native protein. A quantitative contribution of the C-terminal domain to interaction strength or specificity nevertheless cannot be excluded and will require domain-mapping studies in future work. The two proteins (GmNFYA11 and GmNFYA14) antagonistically modulate lipid synthesis and form a bidirectional module controlling oil accumulation, offering a theoretical basis and gene resources for optimizing soybean lipid networks and high-oil molecular breeding. *GmNFYA11* positively regulates oil synthesis through two pathways: it activates transcription of the lipid synthesis gene *GmOLEO1*, and interacts with GmWRI1a to strengthen its transcriptional activity, thereby raising oil and fatty acid levels in seeds. Its elite haplotype Hap02 has been utilized in high-oil soybean breeding (Gou et al., 2026). Conversely, GmNFYA14 is a negative regulator whose interaction with GmWRI1a weakens the latter’s promoter-binding and transcriptional activation abilities, repressing fatty acid and TAG synthesis as well as lipid pathway gene expression, and ultimately lowering seed oil content. Given their opposite functions toward the same target, we propose a working model in which GmNFYA11 and GmNFYA14 compete for GmWRI1a binding to maintain lipid synthesis homeostasis; however, this model remains hypothetical, as direct competition between the two proteins has not yet been experimentally demonstrated. Future experiments, such as competitive pull-down or co-immunoprecipitation assays, together with quantitative binding-affinity measurements, will be required to validate this model. GmNFYA14 physically interacts with both GmLEC1 and GmWRI1a. Further research is required to verify the interaction between GmNFYA11 and GmLEC1, and to clarify whether GmNFYA11 and GmNFYA14 compete for binding to GmLEC1 to alter its regulatory activity and thereby modulate lipid accumulation in soybean. This hypothesis likewise awaits direct experimental validation. This unconfirmed mechanism is important for understanding functional divergence within the NF-YA subfamily and refining the regulatory network of soybean lipid metabolism.

### 4.3 Functional differentiation and haplotype variation of GmNFYA14

In plants, a single NFYA regulatory factor usually possesses multiple biological functions.*AtNFYA5* positively mediates drought resistance by regulating stomatal movement through miR169, and is also involved in multiple growth and developmental processes including seed germination, seedling growth and flowering time (Li et al., 2008; Mu et al., 2013). In rice, *OsNFYA3* directly activates gibberellin (GA) biosynthesis genes to promote plant growth; meanwhile, it binds to and activates abscisic acid (ABA) catabolic genes to attenuate ABA signaling, thereby reducing osmotic stress tolerance (Jin et al., 2023). *GmNFYA14*, characterized in this study, serves as a typical example of such multifunctional regulators. In addition to modulating seed oil biosynthesis, previous studies have demonstrated that *GmNFYA14* positively regulates seed protein content and seed weight, and negatively participates in salt-stress response in soybean (Wang et al., 2023a; Zhang et al., 2025a). This study mainly dissects the molecular mechanism underlying its function in seed oil accumulation. Zhang et al. (2025a) reported peak *SW14* expression at ~21 DAF, whereas **Supplementary Figure 4A** shows a peak at ~42 d after flowering. This likely reflects differences in growth conditions and staging conventions. Both studies nevertheless agree that *GmNFYA14*/*SW14* is seed-expressed and negatively regulates oil accumulation. Consistent with this role, systematic field agronomic evaluation of these lines remains to be performed; nevertheless, we consider it unlikely that the oil phenotypes observed here reflect gross developmental penalties. First, the *sw14* mutants (in the same W82 background as our *GmNFYA14_KO* lines; Zhang et al., 2025a) showed no significant differences from wild type in plant height, node number, branch number, or seed number per plant in replicated field trials at two latitudes, with only 100-seed weight and protein content reduced. Second, the phenotype of the *GmNFYA14*-overexpressing line likewise parallels the naturally occurring *SW14*_H3 haplotype, which increased 100-seed weight and protein content without affecting other agronomic traits (Zhang et al., 2025a). The trade-off between oil and protein contents implies that the breeding value of modulating GmNFYA14 depends, to some extent, on the target market. Notably, given that the repression of oil accumulation is mediated mainly through the GmWRI1a branch, whereas the effects on seed weight and protein content are at least partly mediated by the GmLEC1-containing NF-Y complex, it may be possible to partially uncouple oil gain from the protein penalty by disrupting only the GmNFYA14–GmWRI1a interaction, a strategy that we believe merits testing in future breeding programs. Transcriptome analysis further revealed that *GmNFYA14* extensively regulates a wide range of biological pathways, including metabolic pathways, plant hormone signal transduction, carbohydrate metabolism and plant stress-related pathways (**Figure 6B**). Three major haplotypes of *SW14* exist in natural soybean populations. Haplotype *SW14*_H3, harboring the Pro80Ser and His87Tyr variants, exhibits enhanced protein stability against 26S proteasome degradation, increasing 100-seed weight and seed protein content while reducing oil content. This elite haplotype underwent artificial selection during soybean domestication, accounting for approximately 48.8% of cultivated soybean accessions (Zhang et al., 2025a). Notably, whether such haplotype variations lead to functional differentiation of *GmNFYA14* in regulating soybean salt tolerance and other biological functions remains to be elucidated. Moreover, *GmNFYA*, a homologous gene of *GmNFYA14*, exhibits opposite regulatory effects and positively promotes oil accumulation in soybean (Lu et al., 2021), implying widespread functional differentiation among *NFYA* family members, whose molecular mechanisms and biological significance warrant in-depth investigation in future studies.

## 5 Conclusions

To explore the molecular mechanism underlying the function of the key lipid regulator *GmWRI1a*, this study identified GmNFYA14 as its interacting protein. The seed oil content, total fatty acid level and TAG accumulation were determined in *GmNFYA14*-overexpressing and knockout transgenic soybean plants. The results demonstrated that *GmNFYA14* negatively regulates soybean oil accumulation by inhibiting the biosynthesis of total fatty acids and TAG. Mechanistic analysis indicated that the interaction between GmNFYA14 and GmWRI1a represses the transcriptional regulatory activity of GmWRI1a on its target gene *GmPKP-β1*, and weakens the binding capacity of GmWRI1a to downstream target promoters. Together, these findings support passive interference rather than active repression: GmNFYA14 inhibits GmWRI1a-dependent transcription without directly binding the tested promoter elements. Transcriptomic analysis further confirmed that overexpression of *GmNFYA14* significantly downregulates the expression of *GmWRI1a*-targeted lipid synthesis-related genes (**Figure 7**). Given the reported pleiotropy of *GmNFYA14*/*SW14* in regulating oil content, protein content, and seed weight (Zhang et al., 2025a), the GmNFYA14–GmWRI1a module should be considered a promising entry point for high-oil breeding. Collectively, this study provides novel insights into the molecular mechanism by which *GmNFYA14* modulates soybean oil accumulation, improves the transcriptional regulatory network of plant lipid metabolism, and offers valuable genetic resources and theoretical references for the molecular improvement of high-oil soybean.

**Figure 7:**
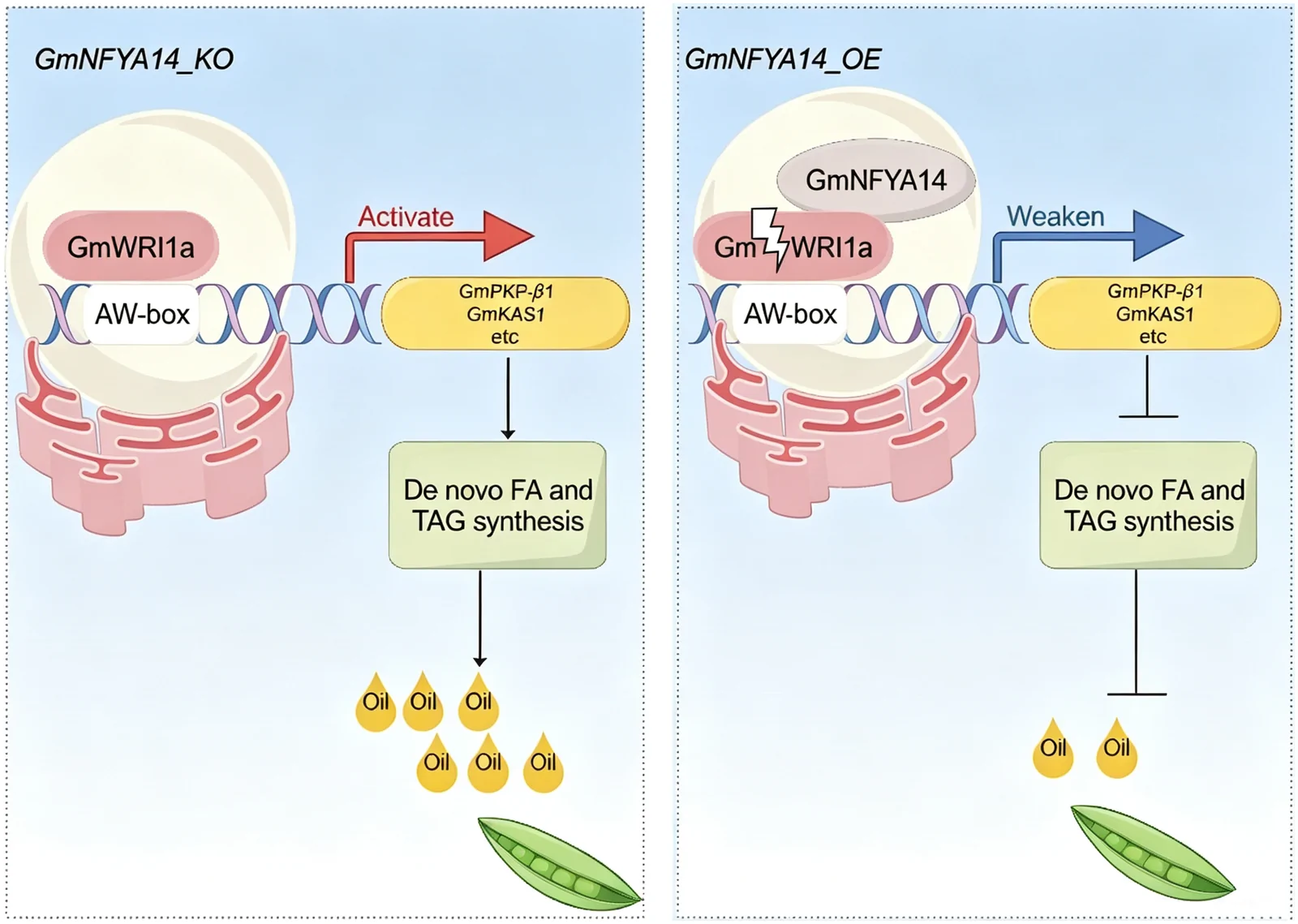
A working model for the regulatory mechanism of soybean oil content mediated by the *GmNFYA14*/*GmWRI1a* module. This model illustrates the regulatory mechanism of oil metabolism mediated by the *GmNFYA14*-*GmWRI1a* module. In *GmNFYA14* knockout lines (*GmNFYA14_KO*), the absence of GmNFYA14 enables GmWRI1a to normally bind to the AW-box motif on the promoters of target genes. This activates the expression of downstream lipid synthesis-related genes and promotes the accumulation of fatty acids and TAG, thereby increasing soybean oil content. In contrast, in *GmNFYA14* overexpression lines (*GmNFYA14_OE*), GmNFYA14 interacts with GmWRI1a. This interaction represses the transcriptional activation and DNA-binding capacity of GmWRI1a, downregulates the expression of downstream lipid synthesis genes, impedes fatty acid and TAG biosynthesis, and ultimately reduces soybean oil accumulation. GmNFYA14 is depicted as a non-DNA-bound inhibitory partner that does not directly bind the AW-box and suppresses GmWRI1a activity through physical interaction with GmWRI1a.

## Data Availability

The RNAseq data generated from this study have been deposited in the Genome Sequence Archive (Zhang et al., 2025b) in National Genomics Data Center (Members and Partners, 2026), China National Center for Bioinformation/Beijing Institute of Genomics, Chinese Academy of Sciences (GSA: CRA049389) that are publicly accessible at https://ngdc.cncb.ac.cn/gsa.
