# Supplementary material for: GmNFYA14 negatively regulates soybean seed oil content by interacting with GmWRI1a
Source: Front Plant Sci. 2026 Oct 28;17:1916663. doi: 10.3389/fpls.2026.1916663 (PMC13751948; doi:10.3389/fpls.2026.1916663)
Supplement: Supplementary file 1 [file DataSheet1.docx]

***Supplementary data***

*GmNFYA14 negatively regulates soybean seed oil content by interacting with GmWRI1a*

Han Gou^a, c^, Xi Li^a^, Shuxin Chai^a^, Meiling Chen^a^, Xiaochao Chen^a, b^, Yunhua Yang^a, c^, Huan Du^a, c^, Bao-Jian Ding^a^, Han Xing^c,^ *, Ping Deng ^a,^ *, Fengjie Yuan^a, b,^ *, Bei Liu^a,^ *

^a^ Xianghu Laboratory, Hangzhou 311200, China

^b^ Zhejiang Academy of Agricultural Sciences, Hangzhou 310021, China

^c^ College of Agriculture, Nanjing Agricultural University, Nanjing 210095, China

* Correspondence: hanx@njau.edu.cn (H.X.); dengping6411@126.com (P.D.); fjyuanhz@126.com (F.Y.); liubei@xhlab.ac.cn (B.L.)

**Fig. S1 Molecular characterization of *GmNFYA14*.** (A) The relative expression levels of *GmNFYA14* in the root, stem, leaf, flower, mature seed and seed of W82 grown for 21, 28, 35, 42, 49 and 56 days after flowering. Leaves, stems and roots from 1-week-old seedlings. Data are presented as means ± SD, with each value derived from three biological replicates. (B) Self-activation assay of *GmNFYA14*. The pGBKT7-GmNFYA14 (BD-GmNFYA14) construct was co-transformed with empty pGADT7 (AD). The combination of pGBKT7 (BD) and pGADT7 (AD) served as the negative control, while pGBKT7-53 (BD-53) paired with pGADT7-T (AD-T) was used as the positive control. (C) Subcellular localization of GmNFYA14 in the *Nicotiana tabacum* leaves. mCherry was used as the nuclear localization signal and 35S::eGFP was used as the control. Bar = 20 μm.

**Fig. S2 Identification of self-activation of *GmWRI1a*.** The recombinant vectors pGBT9-GmWRI1a and pGBT9-GmWRI1a-NT (C-terminal deletion) were separately transformed into Y2HGold competent cells, and the bacterial solutions were spread onto SD/-Trp and SD/-Trp/AbA800 medium. pGBT9-GmWRI1a showed self-activation activity, while pGBT9-GmWRI1a-NT failed to grow on SD/-Trp/AbA800 medium, suggesting that its self-activation was inhibited.

**Fig. S3 Molecular detection of *GmNFYA14_OE* soybean plants.** (A) Glufosinate-ammonium of *GmNFYA14_OE* and W82 soybean leaves. The leaf of soybean seedlings was applied with glufosinate-ammonium. These leaves were observed after 3 days. Glufosinate concentration is 200 mg/L. Scale bars, 2 cm. Detection analysis of the *Bar* gene (B) expression in *GmNFYA14_OE* and W82 soybean leaves.

**Fig. S4 Schematic diagram of the promoter region of the GmWRI1a target gene used for EMSA.** Predicted GmWRI1a binding sites (Probe1–Probe4) are indicated.

**Fig. S5 RT‑qPCR was performed to detect the transcript levels of DEGs between *GmNFYA14_OE48* and W82 seeds in seeds of wild-type W82 and *GmNFYA14*-overexpressing line OE48 and OE49. Data are presented as mean ± SD from three biological replicates (n = 3); error bars denote SD.**

**Table S1** List of primers used in this study.

|  | **Experiment** | **Name** | **Forward (5’-3’)** | **Reverse (5’-3’)** |
| --- | --- | --- | --- | --- |
|  | Yeast two-hybrid | pGBT9-GmWRI1a-NT | TTGACTGTATCGCCGGAATTCATGAAGAGGTCTCCAGCATC | TGGCTGCAGGTCGACGGATCCTCAAGCCAAGTTTGGATCTT |
|  |  | pGBKT7-GmNFYA14 | CATGGAGGCCGAATTCATGCAATCCAAATCTGAAAC | GGATCCCCGGGAATTCTTACTGAATAGCAAGACGCC |
|  |  | pGADT7-GmNFYA14 | GCCATGGAGGCCAGTGAATTCATGCAATCCAAATCTGAAAC | ATGCCCACCCGGGTGGAATTCTTACTGAATAGCAAGACGCC |
| Luciferase complementation assay | | pCAMBIA1300-nLUC-GmWRI1a | CGGGGGACGAGCTCGGTACCATGAAGAGGTCTCCAGCATC | TTTGTTGGATCCCGGGTCATAGATCTAGAGCATAGT |
|  |  | pCAMBIA1300-cLUC-GmNFYA14 | ACGCGTCCCGGGGCGGTACCATGCAATCCAAATCTGAAAC | GTAGTCCATTTGTTGGATCCTTACTGAATAGCAAGACGCC |
|  | Bimolecular fluorescence complementation | pSUPER-nYFP-GmWRI1a | ATACACCAAATCGACTCTAGAATGAAGAGGTCTCCAGCATCTTCT | TTGCTCCATGAGCTCGGTACCTAGATCTAGAGCATAGTCACAAGAAACTG |
|  |  | pSUPER-cYFP-GmNFYA14 | AAGCTTCTGCAGGGGCCCGGGATGCAATCCAAATCTGAAAC | GGGAAATTCGAGCTCGGTACCTTACTGAATAGCAAGACGCC |
|  | GST Pull-Down | pGEX-6P-1-GmNFYA14 | TTCCAGGGGCCCCTGGGATCCATGCAATCCAAATCTGAAAC | GTCACGATGCGGCCGCTCGAGTTACTGAATAGCAAGACGCCTCT |
|  |  | pET-32a-GmWRI1a | GCCATGGCTGATATCGGATCCATGAAGAGGTCTCCAGCATCTTCT | ACGGAGCTCGAATTCGGATCCTCATAGATCTAGAGCATAGTCACAAGAAA |
|  | Co-IP | pYFP-BAM3-GmWRI1a | ATGGCAGGCCTTACTAGTATGAAGAGGTCTCCAGCATC | CTTGCTCACCATGGATCCTAGATCTAGAGCATAGTCAC |
|  |  | pHA-SFT-GmNFYA14 | GCAGGCCTTACTAGTGGATCCATGCAATCCAAATCTGAAAC | AACATCGTATGGGTACCCGGGCTGAATAGCAAGACGCCTCT |
|  | EMSA | pET-32a-GmNFYA14 | GCCATGGCTGATATCGGATCCATGCAATCCAAATCTGAAAC | ACGGAGCTCGAATTCGGATCCTTACTGAATAGCAAGACGCC |
|  |  | Hot probe 1 | TTGGCCTTTGATGTAACCGATTACGTTGCCTTCATCGTCGAC | GTCGACGATGAAGGCAACGTAATCGGTTACATCAAAGGCCAA |
|  |  | Hot probe 2 | TTCTGTTCTGCTTTGCATTCTCCGCCATCATCAT | ATGATGATGGCGGAGAATGCAAAGCAGAACAGAA |
|  |  | Hot probe 3 | AAGTTGTTTGCTTTGGAAGCATCGCTGACAGACA | TGTCTGTCAGCGATGCTTCCAAAGCAAACAACTT |
|  |  | Hot probe 4 | AGTACGCTTCGAACGTAACGCTGAT | ATCAGCGTTACGTTCGAAGCGTACT |
|  | Subcellular localization | pBinGFP4-GmNFYA14 | ACCCCCGGGGTCGACGGATCCATGCAATCCAAATCTGAAAC | GCCCTTGCTCACCATGGATCCCTGAATAGCAAGACGCCTCT |
|  | Generation of transgenic plants | pBA002-GmNFYA14-3Flag | GAGAACACGGGGGACTCTAGAATGCAATCCAAATCTGAAAC | ATCTTTGTAATCCATCTCGAGCTGAATAGCAAGACGCCTCT |
|  | Gene editing | GmNFYA14-sg1 | GGATTGCACTGAATACCTCGCCACCA | AAACTGGTGGCGAGGTATTCAGTGCA |
|  | Quantitative real-time PCR | qGmNFYA14 | GTGCAACAAGGTGCAAGAGG | ACCTGAACAGTCCCCTTCCT |
|  |  | qGmActin11 | GGTGGTTCTATCTTGGCATC | CTTTCGCTTCAATAACCCTA |
|  |  | qGmPKP-β1 | CCACTCGATCCATTCACTCC | TTGTTCCTTTGTGCTCCGAA |
|  |  | qGmKAS1 | AAAAAGGCCCTCGAAAACGC | TCTCCGAATATGGTTCGCCG |
|  |  | qGmKASⅢ-2 | ACCGGTCCCCATATTCATGC | CCGAACCCCAAGTAAGACCC |
|  |  | qGmACBP1 | GCCACCGACTCAATCAAACG | GCTTCCAGCAGCTGTTTCAC |
|  |  | qGlyma.07G019100 | TCCCTCTTCATCAACGCACA | GCTCCACCGAGATTTCCTGT |
|  |  | qGlyma.10G221200 | TCTGCAGTGGCTTCGGATTT | AGGTGAATCGGCCAAGAAGG |
|  |  | qGlyma.11G017900 | ATCGAGGTCGAGAGAGCGAA | CCTGGCTTCCCATCCACAAT |
|  |  | qGlyma.13G162600 | CCCAAAGGACCAAGAACGTC | CTTGTACTCCATCAGCCCCG |
|  |  | qGlyma.16G052000 | TGGACCTGGTGACCGAGAAT | GTGTCCGATCACGACGAACT |
|  |  | qGlyma.19G132900 | AAGGCAACCAACCAAGGGAG | TGGTTCCACAGGAACTGCTA |
|  | LUC dual luciferase  report | pGreenII 0800-proGmPKP-β1 | GTCGACGGTATCGATAAGCTTGGATTAAGTACTTGGAAACG | CGCTCTAGAACTAGTGGATCCTGTTAGAAAGAGAGAGAGAG |
|  |  | pGreenII 62-GmWRI1a | CGCTCTAGAACTAGTGGATCCATGAAGAGGTCTCCAGC | GATAAGCTTGATATCGAATTCTCATAGATCTAGAGCAT |
|  |  | pGreenII 62-GmNFYA14 | CGCTCTAGAACTAGTGGATCCATGCAATCCAAATCTGAAAC | GATAAGCTTGATATCGAATTCTTACTGAATAGCAAGACGCC |

**Table S2** Results of sequencing data quality preprocessing

| Sample | Raw bases (G) | Clean bases (G) | Valid bases (%) | Q30 (%) | GC (%) |
| --- | --- | --- | --- | --- | --- |
| GmNFYA14_OE48_1 | 6.30 | 6.09 | 96.62 | 99.36 | 44 |
| GmNFYA14_OE48_2 | 6.67 | 6.41 | 96.15 | 99.37 | 44 |
| GmNFYA14_OE48_3 | 6.39 | 6.18 | 96.77 | 99.36 | 44 |
| W82_1 | 6.32 | 6.10 | 96.54 | 99.36 | 43 |
| W82_2 | 6.39 | 6.14 | 96.16 | 99.36 | 43 |
| W82_3 | 6.43 | 6.30 | 96.36 | 99.36 | 43 |

GmNFYA14_OE48_1, GmNFYA14_OE48_2, and GmNFYA14_OE48_3 represent three replicates of the *GmNFYA14*_*OE48* line; W82_1, W82_2, and W82_3 represent three replicates of the transgenic receptor W82 as the wild type.

**Table S3** Results of comparison with the reference genome

| Sample | Total reads | Total mapped reads | Ratio (Total mapped reads/Total reads) % |
| --- | --- | --- | --- |
| GmNFYA14_OE48_1 | 40604228 | 39749123 | 97.89 |
| GmNFYA14_OE48_2 | 42724356 | 41816432 | 97.87 |
| GmNFYA14_OE48_3 | 41197752 | 40325823 | 97.88 |
| W82_1 | 40649298 | 39906981 | 96.14 |
| W82_2 | 40949668 | 40186901 | 95.73 |
| W82_3 | 41327378 | 40558788 | 94.91 |

GmNFYA14_OE48_1, GmNFYA14_OE48_2, and GmNFYA14_OE48_3 represent three replicates of the *GmNFYA14*_*OE48* line; W82_1, W82_2, and W82_3 represent three replicates of the transgenic receptor W82 as the wild type.

**Table S4** Results of *GmNFYA14_OE* oil content (%)

| Sample | W82 | OE48 | OE49 | OE50 |
| --- | --- | --- | --- | --- |
| Repeat 1 | 22.79 | 18.15 | 19.70 | 19.71 |
| Repeat 2 | 22.85 | 19.24 | 19.90 | 19.67 |
| Repeat 3 | 22.68 | 18.53 | 19.84 | 19.46 |
| Mean | 22.77 | 18.64 | 19.81 | 19.61 |
| SD | 0.09 | 0.55 | 0.10 | 0.13 |
| *P* value | 1.000000 | 0.000216 | 0.000003 | 0.000004 |

Data are from three biological replicates per genotype (n = 3). Each replicate was a pooled seed sample from 3–5 plants of the same genotype grown in the same batch; plants were not treated as replicates. Values are mean ± SD. *P* values are from two-tailed Student's *t*-test (Section 2.13).

**Table S5** Results of *GmNFYA14_OE* protein content (%)

| Sample | W82 | OE48 | OE49 | OE50 |
| --- | --- | --- | --- | --- |
| Repeat 1 | 36.99 | 47.83 | 41.84 | 41.36 |
| Repeat 2 | 36.94 | 40.08 | 43.27 | 41.37 |
| Repeat 3 | 37.26 | 43.20 | 41.49 | 39.04 |
| Mean | 37.06 | 43.70 | 42.20 | 40.59 |
| SD | 0.17 | 3.90 | 0.94 | 1.34 |
| *P* value | 1.000000 | 0.042117 | 0.000750 | 0.010711 |

Data are from three biological replicates per genotype (n = 3). Each replicate was a pooled seed sample from 3–5 plants of the same genotype grown in the same batch; plants were not treated as replicates. Values are mean ± SD. *P* values are from two-tailed Student's *t*-test (Section 2.13).

**Table S6** Results of *GmNFYA14_KO* TAG content (mg/g)

| Sample | W82 | KO69 |
| --- | --- | --- |
| Repeat 1 | 72.825 | 91.556 |
| Repeat 2 | 72.527 | 83.196 |
| Repeat 3 | 74.389 | 79.775 |
| Mean | 73.250 | 84.840 |
| SD | 1.000 | 6.010 |
| *P* value | 1.000 | 0.031 |

Data are from three biological replicates per genotype (n = 3). Each replicate was a pooled seed sample from 3–5 plants of the same genotype grown in the same batch; plants were not treated as replicates. Values are mean ± SD. *P* values are from two-tailed Student's *t*-test (Section 2.13).

**Table S7** Results of *GmNFYA14_OE* TAG content (mg/g)

| Sample | W82 | OE48 | OE49 |
| --- | --- | --- | --- |
| Repeat 1 | 75.787 | 72.683 | 68.504 |
| Repeat 2 | 76.522 | 69.382 | 65.572 |
| Repeat 3 | 74.338 | 70.258 | 72.459 |
| Mean | 75.550 | 70.770 | 68.840 |
| SD | 1.110 | 1.700 | 3.460 |
| *P* value | 1.000 | 0.015 | 0.033 |

Data are from three biological replicates per genotype (n = 3). Each replicate was a pooled seed sample from 3–5 plants of the same genotype grown in the same batch; plants were not treated as replicates. Values are mean ± SD. *P* values are from two-tailed Student's *t*-test (Section 2.13).

**Table S8** Results of *GmNFYA14_KO* total fatty acid content (mg/g DW)

| Sample | W82 | KO69 |
| --- | --- | --- |
| Repeat 1 | 103.690 | 118.106 |
| Repeat 2 | 103.783 | 117.803 |
| Repeat 3 | 104.880 | 119.085 |
| Mean | 104.120 | 118.330 |
| SD | 0.670 | 0.660 |
| *P* value | 1.00000 | 0.00001 |

Data are from three biological replicates per genotype (n = 3). Each replicate was a pooled seed sample from 3–5 plants of the same genotype grown in the same batch; plants were not treated as replicates. Values are mean ± SD. *P* values are from two-tailed Student's *t*-test (Section 2.13).

**Table S9** Results of *GmNFYA14_OE* total fatty acid content (mg/g DW)

| Sample | W82 | OE48 | OE49 |
| --- | --- | --- | --- |
| Repeat 1 | 113.442 | 101.217 | 101.627 |
| Repeat 2 | 112.955 | 101.272 | 101.776 |
| Repeat 3 | 113.563 | 101.374 | 102.321 |
| Mean | 113.320 | 101.290 | 101.910 |
| SD | 0.320 | 0.080 | 0.370 |
| *P* value | 1.00000000 | 0.00000038 | 0.00000220 |

Data are from three biological replicates per genotype (n = 3). Each replicate was a pooled seed sample from 3–5 plants of the same genotype grown in the same batch; plants were not treated as replicates. Values are mean ± SD. *P* values are from two-tailed Student's *t*-test (Section 2.13).

**Table S10** Results of *GmNFYA14_OE* fatty acid composition (%)

| Sample | W82 | | | OE48 | | | OE49 | | | Mean | | | SD | | | *P* value | |
| --- | --- | --- | --- | --- | --- | --- | --- | --- | --- | --- | --- | --- | --- | --- | --- | --- | --- |
|  | Repeat 1 | Repeat 2 | Repeat 3 | Repeat 1 | Repeat 2 | Repeat 3 | Repeat 1 | Repeat 2 | Repeat 3 | W82 | OE48 | OE49 | W82 | OE48 | OE49 | OE48 | OE49 |
| 16:0 | 13.055 | 13.088 | 13.017 | 12.578 | 12.542 | 12.563 | 12.447 | 12.438 | 12.441 | 13.054 | 12.563 | 12.441 | 0.036 | 0.017 | 0.006 | 0.000028708 | 0.000007874 |
| 18:0 | 3.236 | 3.345 | 3.122 | 3.158 | 3.157 | 3.156 | 3.288 | 3.288 | 3.273 | 3.235 | 3.157 | 3.283 | 0.112 | 0.001 | 0.028 | 0.294414672 | 0.49433212 |
| 18:1 | 18.586 | 18.624 | 18.579 | 19.184 | 19.127 | 19.136 | 21.701 | 21.735 | 21.754 | 18.596 | 19.149 | 21.730 | 0.024 | 0.031 | 0.027 | 0.000015860 | 0.000000012 |
| 18:2 | 53.146 | 52.996 | 53.180 | 52.684 | 52.764 | 52.746 | 51.645 | 51.651 | 51.627 | 53.107 | 52.731 | 51.641 | 0.096 | 0.042 | 0.012 | 0.003611390 | 0.000827545 |
| 18:3 | 10.579 | 10.635 | 10.614 | 11.205 | 10.955 | 10.505 | 9.603 | 9.585 | 9.607 | 10.609 | 10.888 | 9.598 | 0.028 | 0.353 | 0.012 | 0.245411253 | 0.003258882 |

*GmNFYA14_OE48* and *GmNFYA14_OE49* represent three lines of the *GmNFYA14*_*OE*; W82 represent the transgenic receptor as the wild type. Data are from three biological replicates per genotype (n = 3). Each replicate was a pooled seed sample from 3–5 plants of the same genotype grown in the same batch; plants were not treated as replicates. Values are mean ± SD. *P* values are from two-tailed Student's *t*-test (Section 2.13).
